# Sirtuin-1 directly binds and deacetylates hepatic PCSK9 thereby promoting the inhibition of LDL receptor degradation

**DOI:** 10.1093/cvr/cvaf087

**Published:** 2025-07-14

**Authors:** Srividya Velagapudi, Melroy X Miranda, Priyanka Adla, Simon Kraler, Shafeeq A Mohammed, Shekhar Baki, Jerome Robert, Lucia Rohrer, Hwan Lee, Hyun-Duk Jang, Slayman Obeid, Anne Tailleux, Bart Staels, Naresh Babu V Sepuri, Francesco Paneni, Ravi Kumar Gutti, Arnold von Eckardstein, Hyo-Soo Kim, Alexander Akhmedov, Giovanni G Camici, Thomas F Lüscher

**Affiliations:** Center for Molecular Cardiology, University of Zürich, Schlieren Campus, Switzerland; Center for Molecular Cardiology, University of Zürich, Schlieren Campus, Switzerland; Department of Biochemistry, School of Life Sciences, University of Hyderabad, Hyderabad, India; Center for Molecular Cardiology, University of Zürich, Schlieren Campus, Switzerland; Center for Translational and Experimental Cardiology (CTEC), Department of Cardiology, Zurich University Hospital and University of Zürich, Zürich, Switzerland; Department of Biochemistry, School of Life Sciences, University of Hyderabad, Hyderabad, India; Institute of Clinical Chemistry, University Hospital Zürich, Zürich, Switzerland; Institute of Clinical Chemistry, University Hospital Zürich, Zürich, Switzerland; Biomedical Research Institute, Seoul National University Hospital, Seoul, S.Korea; Biomedical Research Institute, Seoul National University Hospital, Seoul, S.Korea; Department of Cardiology, University Hospital Zürich, Zürich, Switzerland; Université Lille, INSERM, Institut Pasteur de Lille, Lille, France; Université Lille, INSERM, Institut Pasteur de Lille, Lille, France; Department of Biochemistry, School of Life Sciences, University of Hyderabad, Hyderabad, India; Center for Translational and Experimental Cardiology (CTEC), Department of Cardiology, Zurich University Hospital and University of Zürich, Zürich, Switzerland; Department of Biochemistry, School of Life Sciences, University of Hyderabad, Hyderabad, India; Institute of Clinical Chemistry, University Hospital Zürich, Zürich, Switzerland; Biomedical Research Institute, Seoul National University Hospital, Seoul, S.Korea; Center for Molecular Cardiology, University of Zürich, Schlieren Campus, Switzerland; Center for Molecular Cardiology, University of Zürich, Schlieren Campus, Switzerland; Center for Molecular Cardiology, University of Zürich, Schlieren Campus, Switzerland; Heart Division, Royal Brompton and Harefield Hospitals GSTT, UK; Cardiovascular Academic Group, King’s College, London, UK

**Keywords:** Sirtuin-1, Post-translational modification, PCSK9, LDL-cholesterol, Hepatic LDLR, Atherosclerosis

## Abstract

**Aims:**

Low-density lipoprotein (LDL)-cholesterol is causally involved in atherosclerotic cardiovascular disease (ASCVD) pathogenesis. Pharmacological activation of the intracellular NAD ^+^ -dependent deacetylase Sirtuin-1 (SIRT1) reduces plasma LDL-cholesterol levels by increasing hepatic LDL-receptor (LDLR) expression, which intriguingly associates with atheroprotective effects. Recent studies have identified the presence of SIRT1 in plasma, however, its effects remain elusive. We found that plasma levels of SIRT1 to be decreased in atherosclerotic mice compared with wild-type controls and aimed to investigate the therapeutic potential of systemic SIRT1 restoration on lipid metabolism and plaque burden in atherosclerotic mice and dissect the underlying molecular mechanisms involved.

**Methods and results:**

Twelve-week-old apolipoprotein E-deficient (*ApoE^−/−^*) mice fed a high-cholesterol diet (1.25% w/w) were randomized to receive recombinant murine SIRT1(rmSIRT1) (*n* = 6; 0.3 mg/kg BW *i.p*.) or vehicle (*n* = 6; PBS) every third day over 4 weeks. Boosting systemic SIRT1 levels increased hepatic LDLR protein expression, reduced plasma LDL-cholesterol levels and decreased plaque progression in *ApoE^−/−^* mice. Yet, rmSIRT1 treatment did not change hepatic proprotein convertase subtilisin/kexin type 9 (PCSK9) expression but notably increased its deacetylated levels. Mechanistically, rmSirt1 directly bound to hepatic PCSK9 thereby promoting PCSK9 deacetylation involving 3 sites, namely Lys243, Lys421, and Lys506, as shown by mass spectrometric analyses. *In vitro* mutagenesis to triple deacetylation mimetic (3KR) reduced SIRT1-induced PCSK9 activity, as evidenced by increased cellular binding and association of ^125^I-LDL to hepatic LDLR. Finally, plasma levels of SIRT1 and PCSK9 were assessed at baseline in patients with acute coronary syndromes. In these patients, plasma SIRT1 levels correlated inversely with PCSK9 with high SIRT1 levels conferring a reduced risk of major adverse cardiovascular events (MACE).

**Conclusion:**

SIRT1 directly binds hepatic PCSK9 and decreases its activity by deacetylation, thereby enhancing LDL-cholesterol clearance by hepatic LDLR upregulation. Boosting circulating SIRT1 exerts atheroprotective effects in mice, with high levels associating with improved prognosis in patients with established ASCVD.

## Graphical Abstract

**Graphical Abstract:**
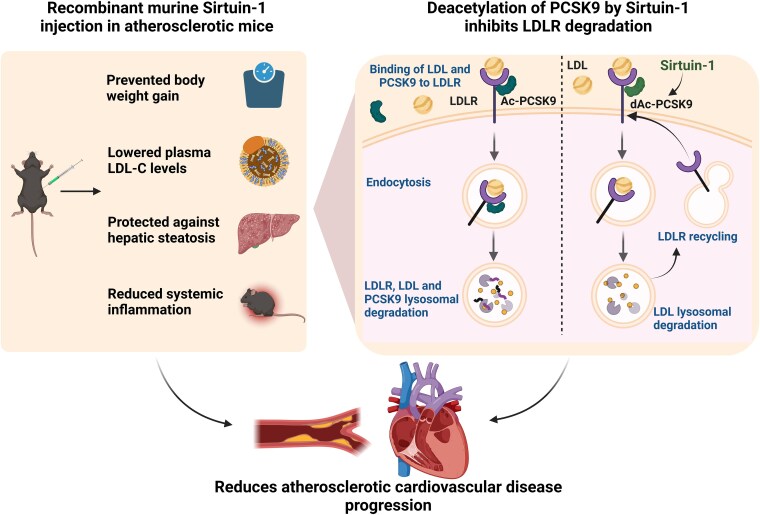
Figure was created with BioRender.com.

**Time of primary review: 40 days**

## 1. Introduction

Proprotein convertase subtilisin/kexin type-9 (PCSK9) increases plasma low-density lipoprotein (LDL) cholesterol (LDL-C) levels by directing hepatic low-density lipoprotein receptor (LDLR) to lysosomes for degradation.^1,2^ Atherosclerotic cardiovascular disease (ASCVD) is a lipid-driven chronic inflammatory condition.^3^ Elevated LDL-C levels are linked to an increased risk of ASCVD,^4,5^ whereas aggressive LDL-C lowering reduces coronary plaque burden and reduces the risk of ASCVD-related events.^6–8^ Indeed, targeted PCSK9 inhibition restores hepatic LDLR levels, decreases plasma LDL-C levels and improves cardiovascular outcomes.^7,9–11^ Importantly, PCSK9 is highly expressed in hepatocytes and is synthesized as a zymogen that undergoes autocatalytic processing in the endoplasmic reticulum.^12,13^ Upon intracellular processing, PCSK9 is secreted from hepatocytes where it binds extracellularly to the epidermal growth factor-A (EGF-A) domain of LDLR leading to its lysosomal degradation.^14,15^ While PCSK9 is known to undergo a series of post-translational modifications,^16^ the effect of post-translationally modified PCSK9 on its hepatic secretion and thus interaction with EGF-A of LDLR remains elusive.

In our previous study,^17^ we provide evidence that pharmacological activation of intracellular Sirtuin-1 (SIRT1) in cultured hepatocytes enhanced hepatic LDLR levels by regulating PCSK9 in a post-translational manner.^17^ Yet, the type of post-translational modification through which SIRT1 deacetylase regulates the activity and function of PCSK9 is largely unknown. More recent reports identified SIRT1 in the circulation,^18,19^ but its role in regulating plasma LDL-C and, in turn ASCVD pathogenesis is unknown. To that end, we investigated the effects and underlying molecular mechanisms exerted by circulating SIRT1 on lipid metabolism, inflammation, and plaque formation in *ApoE^−/−^* mice fed on high-cholesterol diet and assessed the translational value of our experimental findings by measuring SIRT1 and PCSK9 in plasma of patients with acute coronary syndrome (ACS), an acute but common complication of ASCVD.

## 2. Methods

### 2.1 Mouse models

All experiments and animal care procedures were approved by the local veterinary authorities and carried out in accordance with our institutional guidelines (ZH293/14). 8-week-old male *ApoE^−/−^* mice on a pure C57BL/6J background were housed with a 12-h light-dark cycle and were fed a high-cholesterol diet containing 1.25% cholesterol (D12108; Research Diets, USA) for 4 weeks. At the age of 12 weeks, the mice were randomized to get injected with either vehicle control (Phosphate buffer saline containing 0.1% BSA) or with recombinant murine Sirtuin-1 protein (rmSIRT1, CSB-MP846058MO, Cusabio Biotech, LubioScience, Switzerland) intraperitoneally every third day for 4-weeks (*n* = 6 per group). The concentration of rmSIRT1 injected was 0.3 mg/kg of mouse body weight. Body weight was recorded at baseline and weekly for the entire treatment period of 4 weeks. At the end of the treatment period, glucose tolerance test (GTT) was performed on mice fasted for 12 h, and blood glucose was measured following 0.5 g/kg of body weight intraperitoneal glucose injection using glucometer (Contour XT, Bayer). Serum sample collected at time point 0 during GTT was used to measure basal insulin levels (using ELISA, ELM-Insulin, RayBio). Following the tests, 16-week-old mice were euthanised by CO_2_ followed by cardiac puncture (exsanguination) after overnight fasting (16 h), EDTA blood was collected, and tissues were harvested.

### 2.2 Cholesterol profile in the different lipoprotein fractions

The plasma sample was loaded on a gel chromatography column, which separates lipoproteins according to their size. The chromatograph effluent was immediately and continuously mixed with cholesterol assay reactant and incubated at the appropriate conditions of temperature and duration to allow the enzymatic reaction to occur. Cholesterol was measured using colorimetric method (BioMérieux, France). The OD measured at the appropriate wavelength was automatically converted into a graphic signal representing the lipid distribution profile. The area under each peak is proportional to the lipid concentration in the respective lipoprotein fraction.

### 2.3 Patient samples

A sub-cohort of 168 patients with ACS were randomly selected from the investigator-driven, multicentre SPUM-ACS study (ClinicalTrials.gov Identifier: NCT01000701), a prospective cohort study involving four PCI-capable university hospitals in Switzerland (see Supplementary material online, *Figure S4*). The design and detailed in- and exclusion criteria have been reported previously.^20–23^ Briefly, subjects aged ≥18 years presenting within 5 days (preferably within 72 h) after pain onset with a main diagnosis of non-ST elevation myocardial infarction, ST-elevation myocardial infarction, or unstable angina were enrolled between 2009 and 2017. Included patients had symptoms compatible with angina pectoris (chest pain, dyspnoea) and fulfilled at least one of the following criteria: (i) ECG ischaemic changes such as persistent or dynamic ST-segment deviation, T-waves inversion, new left bundle branch block; (ii) evidence of positive conventional or high-sensitive troponin by local laboratory reference values with a rise and/or fall in enzyme levels; (iii) known CHD defined by pre-existing MI, coronary artery bypass graft, or percutaneous coronary intervention or documented ≥50% stenosis of coronary artery in a previous angiography.^24^ Exclusion criteria comprised severe physical disability, inability to give consent (dementia), and life expectancy <1 year (for non-cardiac reason). All patients were followed over 1 year, and major adverse cardiovascular events (MACE) (composite measure of non-fatal myocardial infarction, non-fatal stroke, ischaemia-driven revascularization, in-stent thrombosis, and death from cardiovascular causes) were adjudicated by an independent clinical endpoint committee. The protocol was approved by the local institutional review board, all study participants gave written informed consent, and all procedures were in accordance with the Declaration of Helsinki.

### 2.4 Cell culture

HuH-7 (hereafter Huh7), human hepatoma cells were cultured in Dulbecco’s modified eagle’s medium (DMEM, Sigma-Aldrich) with 5% foetal bovine serum, 100 U/mL of penicillin and 100 µg/mL streptomycin (Sigma-Aldrich) at 37°C in 5% CO_2_, 95% air incubator. Where indicated, cells were treated with vehicle control (Phosphate buffer saline containing 0.1% BSA) or recombinant human SIRT1 (rhSIRT1, 1 μmol/L) (CSB-EP822202HU, Cusabio Biotech, LubioScience, Switzerland) or recombinant human PCSK9 (rhPCSK9, ab198471, Abcam, concentrations in Figure legends) for 2 h.

### 2.5 Lipoprotein isolation and labelling

LDL (1.019 < d < 1.063 kg/L) was isolated from fresh human normolipidemic plasma of blood donors by sequential ultracentrifugation as described previously.^25,26^ LDL was radioiodinated with Na^125^I by the McFarlane monochloride procedure modified for lipoproteins.^26,27^ Specific activities between 300–900cpm/ng of protein was obtained.

### 2.6 Small interfering RNA transfection

Huh7 cells were reverse transfected with small interfering RNA (Silencer Select, Thermo Fisher Scientific) targeted to LDLR (cat. No. s224006, s224007, s4) or non-silencing control (cat. No. 4390843) at a final concentration of 5 nmol/L using Lipofectamine RNAiMAX transfection reagent (Invitrogen, 13778150) in an antibiotic-free medium. All experiments were performed 72 h post-transfection and transfection efficiency was confirmed using quantitative RT-PCR and Western blotting.

### 2.7 Plasmids and site-directed mutagenesis

Plasmids carrying *PCSK9* mutant variants constructed by PolyGene (Rümlang, Switzerland) were transfected using Lipofectamine 3000 following manufacturers protocol. Cells were harvested 72 h post-transfection.

### 2.8 Lipoprotein binding and cell association

All assays were performed in DMEM (Sigma) containing 25 mmol/L HEPES and 0.2% BSA instead of serum. Huh7 cells were incubated with 10 µg protein/mL of ^125^I-LDL without (total) or with 40 times excess of non-labelled LDL (unspecific) for 2 h at 4°C for cellular binding and at 37°C for cellular association experiments. Specific cellular binding/association was calculated by subtracting the values obtained in the presence of excess unlabelled LDL (unspecific) from those obtained in the absence of unlabelled LDL (total). Specific cellular binding represents the bound ^125^I-LDL specifically to the Huh7 cells at 4°C. Specific cellular association represents both the cellular bound and internalized ^125^I-LDL specifically by the Huh7 cells at 37°C.

### 2.9 Real-time quantitative PCR

Total RNA was isolated using TRI reagent (Sigma T9424) according to the manufacturer’s instruction. Genomic DNA was removed by digestion using recombinant DNase I (Roche) and RNase inhibitor (Ribolock, Thermo Scientific). Reverse transcription was performed using M-MLVRT (Invitrogen, 200 U/µL) according to the standard protocol provided by the manufacturer. Quantitative PCR was done with LightCycler FastStart DNA Master SYBR Green I (Roche) using gene-specific primers: *LDLR* (For: AAG GAC ACA GCA CAC AAC CA; Rev: CAT TTC CTC TGC CAG CAA CG), normalized to *GAPDH* (For: CCC ATG TTC GTC ATG GGT GT; Rev: TGG TCA TGA GTC CTT CCA CGA TA).

### 2.10 Deacetylation assay

RhPCSK9 (50μg/mL) was incubated with recombinant human SIRT1 (100μg/mL) in a buffer (250 mM Tris–HCl, 20 mM MgCl_2_, 250 mM NaCl) in the presence and absence of 5 mM NAD^+^ for 2 h at 37°C. The reaction was stopped using Laemmeli buffer and subjected to western blotting.

### 2.11 LDLR and PCSK9 binding assay

PCSK9-LDLR interaction was assessed using a CircuLex PCSK9-LDLR *in vitro* binding assay kit (MBL International, Woburn, MA, USA) with minor modifications. RhPCSK9 at a concentration of 50μg/mL was subjected to a deacetylation assay for 2 h at room temperature with gentle shaking. The mixtures were added to an ELISA plate that was coated with EGF-AB peptide of the LDLR. Subsequent procedures were performed according to the manufacturer’s instructions.

### 2.12 Immunoprecipitation

20μg of PCSK9 antibody (RnD Systems) was covalently linked to beaded agarose resin (40μL) via primary amines using the Aminolink Plus immobilization kit (Pierce, catalogue number 44894). The beads were washed five times with PBS + 0.1% NP-40 + protease and phosphatase inhibitors (Roche) and were used for Western blotting analysis.

### 2.13 Western blotting

Huh7 cells and mice liver tissue samples were lysed in RIPA buffer [10 mmol/L Tris pH 7.4, 150 mmol/L NaCl, 1% NP-40, 1% sodium deoxycholate, 0.1% SDS, with protease and phosphatase inhibitors (complete EDTA, Roche)]. Amount of protein in the lysed samples is quantified using Pierce BCA protein assay kit (Thermo Fisher Scientific) 30μg of protein were separated on 8–10% SDS-PAGE and trans-blotted onto PVDF membrane (GE Healthcare). Membranes were blocked in appropriate blocking buffer recommended for the antibody (PBS-T supplemented with 5% milk or BSA) and incubated overnight on shaker at 4°C with primary antibodies in the same blocking buffer; LDLR (1:1000 dilution, ab52818, Abcam), PCSK9 expression in Huh7 cells (1:1000 dilution, ab181142, Abcam), PCSK9 in mouse lysate (1:1000 dilution, AF3985, R&D systems). Membranes were incubated for 1 h with HRP-conjugated secondary antibody (Southern Biotechnology) in blocking buffer. Membranes were further incubated with chemiluminescence substrate for 1 min (Pierce ECL plus, Thermo Fisher Scientific) and imaged using Fusion Fx (Vilber). Where indicated, either Vinculin (1: 10 000 dilution, V4505, Merck) or β-actin (1:2500, ab8226, Abcam, incubation for 1 h at room temperature) was used as loading control.

### 2.14 ELISA

Mice were fasted overnight before blood was drawn prior to harvesting. For plasma analyses EDTA plasma was separated from corpuscular elements by centrifugation at 4°C immediately after blood was drawn and stored at −80°C until further analysis. Deacetylase inhibitors were added to aliquots of mouse plasma for acetylation measurement. ELISA for mouse SIRT1 (no dilution, ab206983, Abcam), mouse RANTES (ELM-RANTES, RayBiotech), and PCSK9 in the plasma were performed using mouse PCSK9 ELISA kit (RnD systems) to assess total PCSK9 and the detection antibody was replaced with acetyl-lysine antibody (9441, CST) for acetyl-PCSK9. ELISA for human SIRT1 (1:10 dilution, LS-F6780, LS Bio) and human PCSK9 (1:10 dilution, DPC900, R&D Systems) were performed following the manufacturers’ protocol (no dilution for cell culture supernatant).

### 2.15 Immunohistochemistry

*En face* plaque analysis was performed on thoraco-abdominal aortae and on liver sections that were fixed overnight with 4% paraformaldehyde (PFA) and then stained with Oil-Red O (ORO). The quantification of the ORO positive area was calculated using Image J. 5μm thick cryosections from the aortic sinus were air-dried, fixed with 4% PFA, and then stained for ORO, anti-CD68 (BioRad, no. MCA1957GA). The quantification of the stained areas was performed with ImageJ. Means were taken by evaluating at least 4 serial cryosections from each mouse.^28^

### 2.16 Fluorescent labelling and uptake studies

rhSIRT1 (1–3 mg) was fluorescently labelled with ATTO-594-NHS-ester dye (ATTO-Tec, AD 594–35). The reaction was performed at a pH 8.0 adjusted by adding 1 M NaHCO3 to obtain a final concentration of 0.1 M in dark at room temperature for 1 h. The labelled rhSIRT1 was separated from free dye by gel filtration chromatography using PD-10 desalting columns (GE Healthcare, 170851-01). All uptake assays were performed in DMEM (Sigma) containing 25 mmol/L HEPES and 0.2% BSA instead of serum. Huh7 cells were cultured in growth medium as monolayers on coverslips. After 72 h, cells were incubated with 1μmol/L of ATTO 594 labelled rhSIRT1 or 1 h at 37°C in dark. Cells were then washed, fixed with 2% PFA and images were acquired on a Zeiss Axiovert 200 M.

### 2.17 Mass spectrometry

Excised gel bands were cut into approximately 1 mm^3^ pieces. Gel pieces were washed twice with 100 mM ammonium bicarbonate/50% acetonitrile for 15 min at 50°C and dehydrated with acetonitrile for 10 min. All supernatants were discarded. Rehydration of the gel pieces was with 10 mM Tris-HCl, 2 mM CaCl_2_, pH 8.2 containing 5 ng/μL proteomics-grade recombinant trypsin (Roche, Diagnostics, Mannheim, Germany) at 4°C. Microwave assisted digestion (Model Discover, CEM, Matthews, NC) was performed for 30 min at 5 W and 60°C. The supernatant was extracted, and gel pieces were washed with 150μL 0.1% trifluoroacetic acid/50% acetonitrile. All supernatants were combined and evaporated to dryness in with a SpeedVac concentrator. Digested samples were re-solubilized in 20μL of 0.1% formic acid and were analysed by either MALDI time-of-flight tandem mass spectrometry MALDI-TOF-TOF) or liquid chromatography electrospray tandem mass spectrometry (LC/MS/MS For Maldi-TOF-TOF 1μL of the sample was mixed with 1μL of matrix solution (0.7 mg/mL α-cyano-4-hydroxycinnamic acid in 0.1% trifluoroacetic acid/85% acetonitrile, 1 mM NH_4_H_2_PO_4_) and spotted on the target, desalted and concentrated by washing the spot with 0.1% trifluoroacetic acid. Maldi spectra were acquired on an UltrafleXtreme (Bruker, Bremen, Germany). LC/MS/MS analyses were run on a nanoAcquity UPLC (Waters Inc.) connected to a Q Exactive mass spectrometer (Thermo Scientific) equipped with a Digital PicoView source (New Objective). An aliquot of 2μL was injected. Peptides were trapped on a Symmetry C18 trap column (5 µm, 180 µm × 20 mm, Waters Inc.) and separated on a BEH300 C18 column (1.7 µm, 75 µm × 150 m, Waters Inc.) at a flow rate of 250 nL/min using a gradient from 1% solvent B (0.1% formic acid in acetonitrile, Romil)/99% solvent A (0.1% formic acid in water, Romil) to 40% solvent B/60% solvent A within 90 min. Full scan MS spectra were acquired in positive profile mode from 350–1500 *m/z* with an automatic gain control target of 3e6, an Orbitrap resolution of 70'000 (at 200 *m/z*), and a maximum injection time of 100 ms. The 12 most intense multiply charged (*z* ≥ + 2) precursor ions from each full scan were selected for higher-energy collisional dissociation fragmentation with a normalized collision energy of 25 (arbitrary unit). Generated fragment ions were scanned with an Orbitrap resolution of 35'000 (at 200 *m/z*) an automatic gain control value of 1e5 and a maximum injection time of 120 ms. The isolation window for precursor ions was set to 2.0 *m/z* and the underfill ratio was at 3.5% (referring to an intensity threshold of 2.9e4). Each fragmented precursor ion was set onto the dynamic exclusion list for 40 s.

Peptides were identified by aligning the MALDI-TOF-TOF data with the known sequence of PCSK9 using the BioTools programme (Bruker, Bremen, Germany) or by searching the SwissProt database (version 2015_11, 549832 entries) using the Mascot search engine (Matrix Science, version 2.4.1). Mascot was set up to search for the SwissProt database assuming the digestion enzyme trypsin. Mascot was searched with a fragment ion mass tolerance of 0.030 Da and a parent ion tolerance of 10.0 PPM. Oxidation of methionine and acetylation of lysine were specified in Mascot as a variable modification. Scaffold (version Scaffold_4.4.8, Proteome Software Inc.) was used to validate MS/MS based peptide and protein identifications. Peptide identifications were accepted if they achieved an FDR less than 0.1% by the Scaffold Local FDR algorithm. Protein identifications were accepted if they achieved an FDR less than 1.0% and contained at least 2 identified peptides.

### 2.18 Statistics

Data are presented as mean ± SEM. Statistical comparisons were done by unpaired Student's *t* test, Mann–Whitney test, one-way analysis of variance (ANOVA), or Kruskal–Wallis H test, as appropriate. A two-tailed *P*-value ≤ 0.05 was deemed statistically significant. At least three independent experiments in triplicates were performed for each experiment. As regards the clinical data, we assumed normality and Pearson correlation coefficients between PCSK9 and SIRT1 plasma levels were calculated, and a linear regression line was fitted. To model the association of SIRT1 tertiles and risk of 1-year MACE, Kaplan-Meier analyses and Cox proportional hazard regression models were employed, with SIRT1 tertiles, sex, age, estimated glomerular filtration rate, and smoking status serving as the independent variables. In sensitivity analyses and to avoid model overfit, the SIRT1-MACE association was tested independently from sex, age, body mass index and a history of hypertension and/or diabetes Statistical analyses were performed using GraphPad Prism 8.2.1 software or R version 4.2.1.

Please see the Major Resources Table for all research materials listed in Section 2.

## 3. Results

### 3.1 rmSIRT1 restores reduced plasma levels and decreases body weight in *ApoE^−/−^* mice

**Figure 1:**
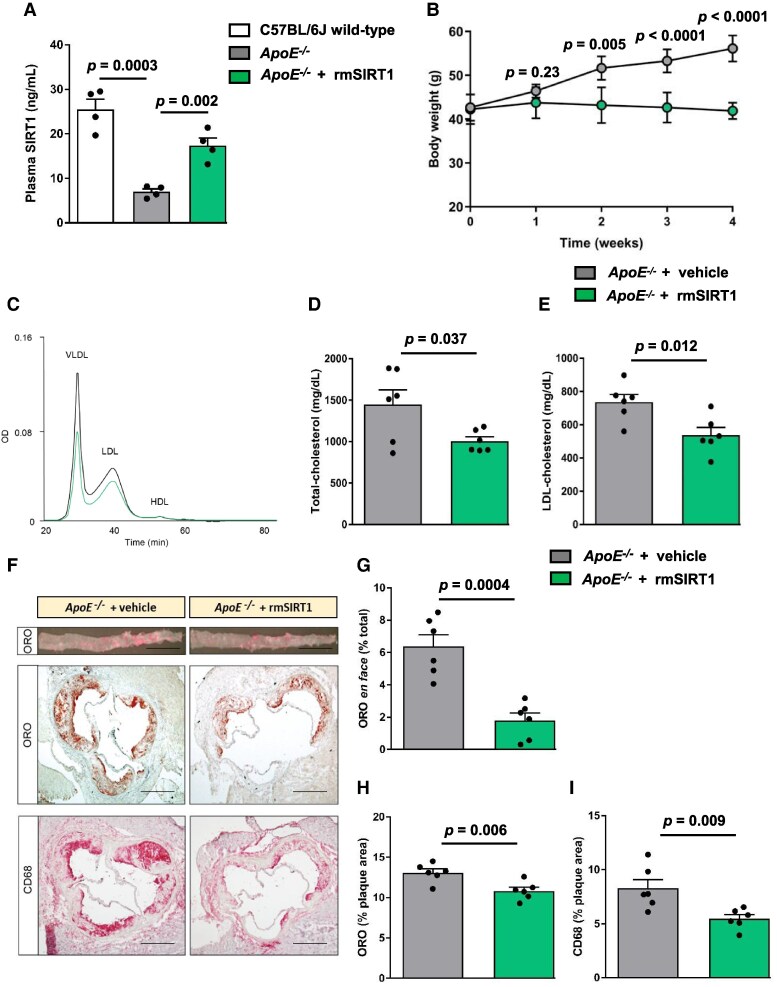
Exogenous SIRT1 treatment reduces plasma LDL-cholesterol and protects against atherosclerosis in *ApoE^−/−^* mice. Eight-week-old *ApoE^−/−^* mice were fed on a high cholesterol diet (1.25% w/w) for 4 weeks and randomized to be treated with rmSIRT1 (*n* = 6) or vehicle (PBS containing 0.1% BSA) (*n* = 6) for another 4 weeks. (*A*) Plasma concentration of SIRT1 measured using ELISA of C57BL/6J wild-type mice (*n* = 6), *ApoE^−/−^* mice + vehicle (*n* = 6) and *ApoE^−/−^* mice + rmSIRT1 (*n* = 6). (*B*) Weekly measurements of body weight of *ApoE^−/−^* mice + vehicle (*n* = 6) and *ApoE^−/−^* mice + rmSIRT1 (*n* = 6). (*C*) Graph showing cholesterol distribution in the different lipoprotein sub-fractions separated by gel filtration chromatography; (*D*) Bar graph of plasma total cholesterol and (*E*) LDL-cholesterol concentrations. (*F*) Representative pictures (left) and quantifications of (*G*) thoracic-abdominal aortae *en face*, (*H*) aortic root cross sections stained with ORO and (*I*) immunohistochemically for macrophages (CD68). Scale bars in photomicrographs: 1 mm (for *E*) and 500 μm (for *F*, *G*). Grey bars represent vehicle treatment and green bars represent rmSIRT1 treatment. ORO, Oil-Red O. Values are represented as means ± SEM. Statistical significance was performed using Student’s unpaired *t*-test.

Plasma levels of circulating SIRT1 were decreased in *ApoE^−/−^* mice fed a high-cholesterol diet (mean ± SEM; 6.988 ± 0.6512 ng/mL, *n* = 4) as compared with wild-type controls (25.48 ± 2.326 ng/mL, *n* = 4, *P =* 0.0003) (*Figure 1A*). Next, 12-week-old *ApoE^−/−^* mice fed on a high-cholesterol diet (for 4-weeks) were randomly assigned to receiving recombinant murine Sirtuin-1 (rmSIRT1, 0.3 mg/kg of mouse body weight, every third day) injections over 4 weeks (rmSIRT1, used as mimetic for circulating SIRT1 protein) (see Scheme Supplementary material online, *Figure S1A*), resulting in restored plasma SIRT1 levels in *ApoE^−/−^* mice (17.35 ± 1.731 ng/mL, *n* = 4, *P* = 0.002) (*Figure 1A*). rmSIRT1 prevented body weight gain (*Figure 1B*), improved glucose tolerance (see Supplementary material online, *Figure S2A* and *B*) and insulin levels (see Supplementary material online, *Figure S2C*) in *ApoE^−/−^* mice.

### 3.2 rmSIRT1 decreases LDL-C, reduces inflammation, and provides athero-protection in *ApoE^−/−^* mice

Notably, treatment of *ApoE^−/−^* mice with rmSIRT1 decreased plasma levels of total cholesterol (*Figure 1C* and *D*), LDL-cholesterol (*Figure 1E*), and VLDL-cholesterol (see Supplementary material online, *Figure S3A*), whereas HDL-cholesterol (see Supplementary material online, *Figure S3B*) levels remained unchanged. Compared with vehicle-treated *ApoE^−/−^* mice, *en face* preparations of the thoracic-abdominal aortae and cross-sections of aortic roots of rmSIRT1-treated *ApoE^−/−^* mice showed reduced plaque size (*Figures 1F–H*) and diminished accumulation of CD68 positive cells (*Figure 1I*). In line, expression of the pro-atherogenic cytokines IL-1β, IL-6, TNF-α and IFN-γ (see Supplementary material online, *Figure S3C*), as assessed using cytokine profiler assay and plasma levels of RANTES, a marker for atherogenesis and fibrosis (see Supplementary material online, *Figure S3D*), were decreased in the plasma of *ApoE^−/−^* mice treated with rmSIRT1. In aggregate, these data indicate that rmSIRT1 delivery restores systemic SIRT1 levels and blunts molecular signatures of atherosclerosis thereby promoting atheroprotective effects in *ApoE^−/−^* mice.

Further, treatment with rmSIRT1 showed decreased lipid deposits in the hepatic sections compared with the vehicle-treated *ApoE^−/−^* mice (see Supplementary material online, *Figure S4A*). Moreover, the levels of hepatic triglycerides (see Supplementary material online, *Figure S4B*) and total cholesterol (see Supplementary material online, *Figure S4C*) were also decreased in the tissue lysates of the mice treated with rmSIRT1. Indeed, rmSIRT1 reduced both hepatic steatosis and atherosclerotic phenotype in the *ApoE^−/−^* mice, therefore our study might be a first step towards establishing circulating SIRT1 as a potent therapeutic against the global lipid burden.

### 3.3 rmSIRT1 increases hepatic LDLR by deacetylation of PCSK9 in *ApoE^−/−^* mice

**Figure 2:**
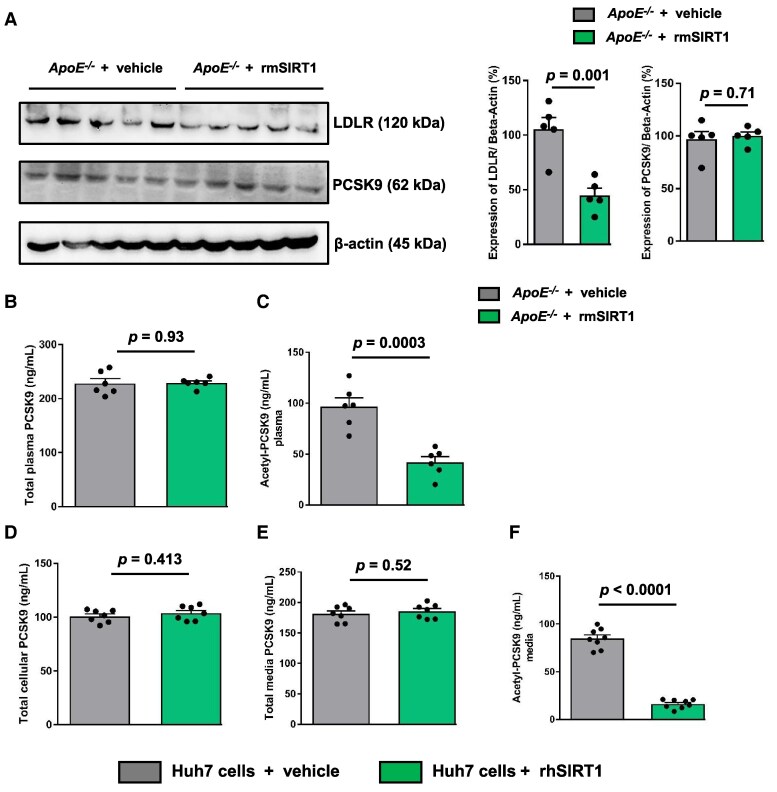
Exogenous SIRT1 increases hepatic LDLR by post-translational modification of PCSK9 in *ApoE^−/−^* mice. Eight-week-old *ApoE^−/−^* mice were fed on a high cholesterol diet (1.25% w/w) for 4 weeks and were randomized to be treated with rmSIRT1 (*n* = 6) or with vehicle (PBS with 0.1% BSA) (*n* = 6) for another 4 weeks. (*A*) Western blot of LDLR (120 kDa) and PCSK9 (∼62 kDa) in hepatic tissue lysates of mice (*n* = 5). β-actin (45 kDa) was used as loading control. Bar graph of ELISA of (*B*) total PCSK9 and (*C*) acetylated PCSK9 in mice plasma. Statistical significance was performed using Student’s unpaired two-sample *t*-test. (*D*, *E*, *F*) Human hepatoma Huh7 cells [three independent triplicate experiments (*n* = 3)] were treated with either 1 μmol/L of recombinant human SIRT1 (rhSIRT1) or vehicle (PBS containing 0.1% BSA) for 2 h at 37°C. The cell culture media was collected, and cells were lysed to perform ELISA to detect PCSK9. Bar graph of ELISA of (*D*) total cellular PCSK9, (*E*) total media PCSK9 and (*F*) acetylated PCSK9 in the media of cultured Huh7 cells. Grey bars represent vehicle treatment and green bars represent rmSIRT1 or rhSIRT1 treatment as indicated. Data are represented as means ± SEM. Statistical significance was performed using Student’s unpaired *t*-test.

Treatment with rmSIRT1 increased the protein levels of SIRT1 in the lysed hepatic tissue as assessed by ELISA, but did not alter its intracellular synthesis (see Supplementary material online, *Figure S5A* and *B*). Notably, this was associated with an increased total hepatic LDLR protein expression relative to vehicle-treated controls (*Figure 2A*). To assess whether rmSIRT1 affects hepatic LDLR expression through PCSK9 protein levels, we assessed hepatic and plasma PCSK9 levels and observed no difference between rmSIRT1− and vehicle-treated mice (*Figure 2A* and *B*). Since SIRT1 regulates the activity of many proteins by deacetylation,^29^ the acetylation status of PCSK9 upon rmSIRT1 treatment was studied using enzyme-linked immunosorbent assay (ELISA). Interestingly, the levels of acetyl-PCSK9 were decreased with rmSIRT1 treatment compared with the vehicle-treated mice (*Figure 2C*).

Next, we treated human hepatoma Huh7 cells with recombinant human Sirtuin-1 (rhSIRT1) protein to mimic the circulating SIRT1 protein. While pre-treatment of Huh7 cells with 1 μmol/L of rhSIRT1 for 2 h did not affect total PCSK9 levels neither in lysed cells nor in the collected cell-culture media (*Figure 2D, E*); however, the levels of acetyl-PCSK9 were reduced in the media of rhSIRT1-treated cells compared with the vehicle-treated Huh7 cells (*Figure 2F*), indicating that SIRT1 may indeed deacetylate PCSK9 in the circulation.

### 3.4 SIRT1 inhibits PCSK9-mediated LDLR degradation in hepatocytes

**Figure 3:**
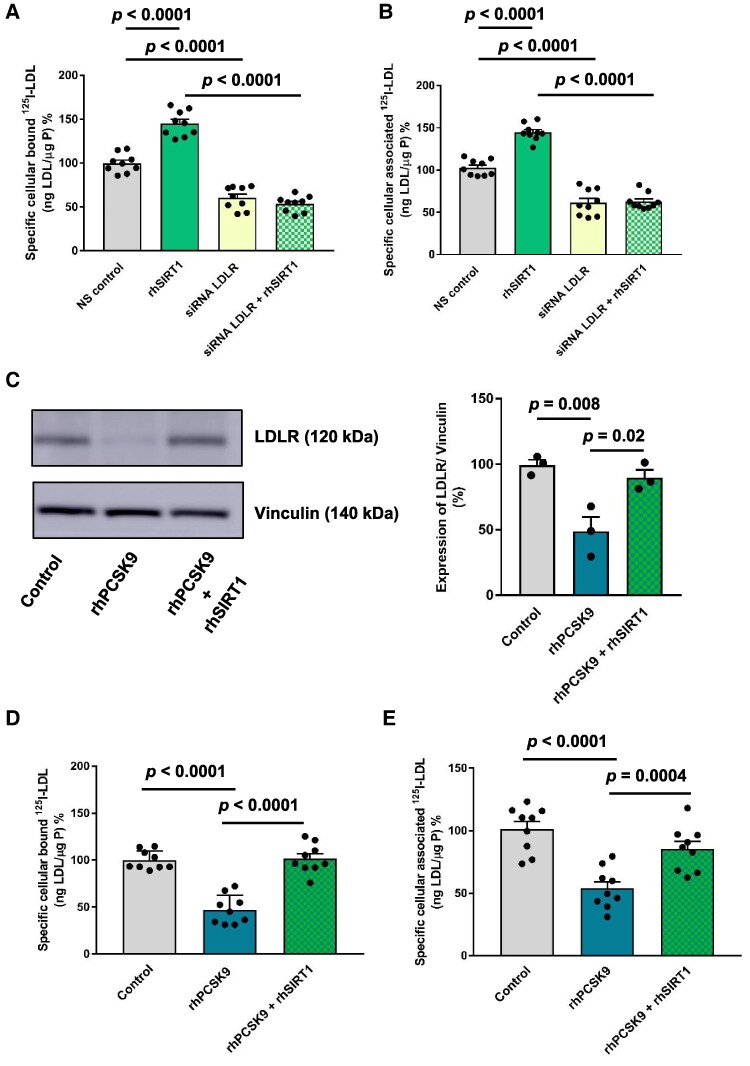
Exogenous SIRT1 inhibits PCSK9-mediated LDLR degradation in hepatocytes. HuH-7 (hereafter Huh7) cells were transfected with a specific siRNA against LDLR or with non-silencing control siRNA (NS control). Assays were performed 72 h post transfection. Cells were then pre-treated with 1 μmol/L of rhSIRT1 for 2 h (*A*) Specific cellular binding of ^125^I-LDL was measured at 4°C (*B*) Specific cellular association of ^125^I-LDL was measured at 37°C. (*C*, *D*, *E*) Huh7 cells were co-incubated with rhPCSK9 (2 μg/mL) with rhSIRT1 (1 μmol/L) for 2 h at 37°C (*C*) Representative Western blots (*n* = 3) and quantification of blot density of LDLR (130 kDa) degradation in Huh7 cells. Vinculin (140 kDa) was used as the loading control. (*D*) Specific cellular binding of ^125^I-LDL was measured at 4°C (*E*) Specific cellular association of ^125^I-LDL was measured at 37°C. Values are represented as means ± SEM of three independent triplicate or more experiments (*n* = 3). Statistical significance was performed using one-way ANOVA followed by Tukey’s multiple comparison test.

To understand whether exogenous SIRT1 decreases plasma LDL-C levels by accelerating hepatic clearance, radio-iodinated LDL uptake studies in Huh7 cells were performed. Pre-treatment of Huh7 cells with 1 μmol/L of rhSIRT1 for 2 h increased the specific cellular association of ^125^I-LDL at 37°C (*Figure 3A*) in Huh7 cells. Next, RNA interference was used to blunt LDLR expression (see Supplementary material online, *Figure S6A* and *B*). Notably, silencing LDLR alone decreased ^125^I-LDL cellular binding and association (*Figure 3A* and *B*). Yet, pre-treatment with 1 μmol/L of rhSIRT1 for 2 h did not restore the cellular binding and association of ^125^I-LDL, inhibited by the suppression of LDLR (*Figure 3A* and *B*). Collectively, these data indicate that SIRT1 increases cellular binding and association of LDL through LDLR in hepatocytes.

To understand whether intracellular uptake of SIRT1 is needed to exert these effects, microscopic uptake studies with ATTO594-rhSIRT1 were performed in Huh7 cells. The data showed that rhSIRT1 is internalised by the Huh7 cells (see Supplementary material online, *Figure S7A*). qRT-PCR studies did not show any change in the endogenous *Sirt1* gene expression, whereas Western Blotting showed that SIRT1 protein levels were increased, indicating these phenotypic effects were not due to the *de novo* synthesis (see Supplementary material online, *Figure S7B*, *S7C*).

Next, treatment of Huh7 cells with rhPCSK9 protein for 2 h decreased total LDLR protein expression in a concentration-dependent manner, indicating LDLR degradation by exogenous rhPCSK9 (see Supplementary material online, *Figure S6C*). Similarly, treatment of Huh7 cells with rhPCSK9 reduced cellular binding and association of ^125^I-LDL in a concentration-dependent manner (see Supplementary material online, *Figure S6D*, *S6E*). Notably, co-incubation of rhPCSK9 (2 μg/mL) with rhSIRT1 (1 μmol/L) in Huh7 cells significantly attenuated LDLR degradation mediated by exogenous PCSK9 (*Figure 3C*). In line with the above, co-incubation of rhPCSK9 with rhSIRT1 restored the specific cellular binding of ^125^I-LDL at 4°C and association of ^125^I-LDL at 37°C (*Figure 3D, 3E*) by Huh7 cells, indicating that SIRT1 decreases the functional activity of PCSK9 and hence prevents hepatic LDLR degradation.

To assess whether the cellularly produced SIRT1 exerts similar effects as exogenously supplemented SIRT1, we treated the cells with inhibitors of secretory pathway, i.e. Brefeldin A and Monensin. We found decreased presence of SIRT1 in the culture medium as assessed by the ELISA (see Supplementary material online, *Figure S7D*), and specific cellular associated ^125^I-LDL in the presence of rhPCSK9 (see Supplementary material online, *Figure S7E*). Whereas incubation with rhSIRT1 in the presence of inhibitors abolished the effects of rhPCSK9, i.e. rhSIRT1 restored the specific cellular association of ^125^I-LDL (see Supplementary material online, *Figure S7D*). These data indicate that the secretion of cellularly produced SIRT1 is important to inactivate PCSK9 and hence clear the circulating LDL-C rather than the endogenous SIRT1.

### 3.5 Circulating SIRT1 directly binds and deacetylates PCSK9

**Figure 4:**
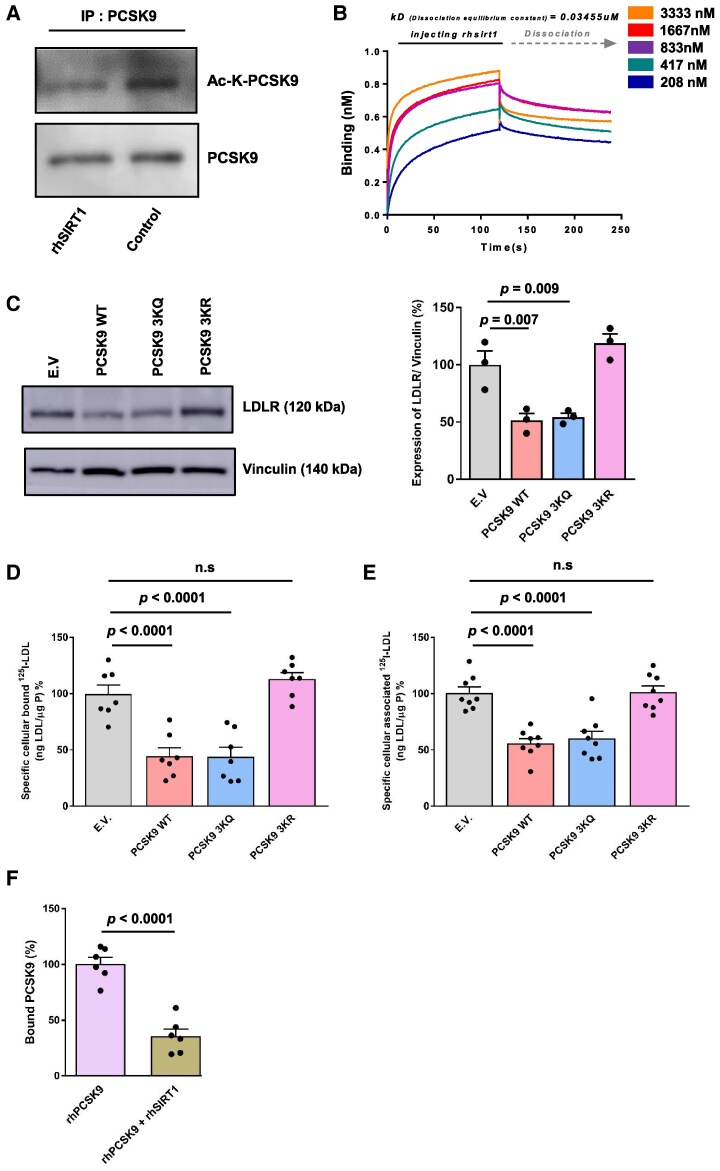
Exogenous SIRT1 inhibits PCSK9 activity through direct deacetylation in hepatocytes—specific mutation of these deacetylation sites restores PCSK9 activity. Huh7 cells were incubated with rhSIRT1 (1 μmol/L) for 2 h at 37°C. (*A*) Western blot analysis of PCSK9 immunoprecipitated Huh7 cells lysates with total PCSK9 antibody (∼62 kDa) and acetyl lysine antibody (Ac-K-PCSK9, ∼62 kDa). (*B*) Direct binding assay interaction between rhSIRT1 and rhPCSK9 using Surface Plasmon Resonance. The equilibrium dissociation constant was 34 nM. (*C*) Schematic diagram of PCSK9 domains and an overview of lysine(K) sites on PCSK9 deacetylated by SIRT1. Representative Western blot and quantification of LDLR (130 kDa) and Vinculin (140 kDa) expression in Huh7 cells expressing WT, 3KR, 3KQ mutants of PCSK9 for 72 h (*n* = 3). *(E*)V. means empty vector (*D*) Specific cellular binding of ^125^I-LDL was measured at 4°C (*E*) Specific cellular association of ^125^I-LDL was measured at 37°C. (*F*) Binding of acetylated and deacetylated PCSK9 to EGF-A of LDLR using ELISA. SP—signal peptide, Pro—pro-domain, Catalytic—catalytic domain, CHRD—cysteine, histidine-rich domain. Values are represented as means ± SEM of three independent triplicate or more experiments (*n* = 3). Statistical significance was performed using one-way ANOVA followed by Tukey’s multiple comparison test.

To delineate the mechanisms through which SIRT1 affects PCSK9 function, we investigated whether SIRT1 deacetylates PCSK9. To that end, Huh7 cells were pre-treated with rhSIRT1 for 2 h, cell lysates were treated with anti-PCSK9 antibody and then pulled down using protein A/G agarose beads. Western blotting analysis with immunoprecipitated protein showed that Huh7 cells pre-treated with rhSIRT1 decreased the expression of acetyl-PCSK9 lysine levels compared with control conditions (*Figure 4A*). To understand whether SIRT1 directly binds to PCSK9, a direct binding assay using Surface Plasmon Resonance (SPR) was performed. The SPR-based assay showed an increase of response units between rhSIRT1 and rhPCSK9 in a dose-dependent manner with an equilibrium dissociation constant of 35 nM, further confirming that PCSK9 is a direct substrate of SIRT1 (*Figure 4B*). Finally, by using mass spectrometry on PCSK9 immunoprecipitated lysates from Huh7 cells, we identified 4 acetylation sites, 3 of which in the catalytic domain (Lys243, Lys273, Lys421) and one in the C-terminal domain (Lys506) (see Supplementary material online, *Figure S8A*). Incubation of Huh7 cells with rhSIRT1 caused deacetylation of PCSK9 on Lys243, Lys421, and Lys506 but not of Lys273 (see Supplementary material online, *Figure S8B*).

### 3.6 Deacetylation of PCSK9 by circulating SIRT1 reduces its activity

To evaluate the functional importance of PCSK9 acetylation, we next generated acetylation and deacetylation mimicking mutants of PCSK9: Substitution of Lys243, Lys421, and Lys506 by glutamine (K243Q/K421Q/K506Q) in PCSK9–3KQ and arginine (K243R/K421R/K506R) in PCSK9–3KR mimic acetylation and deacetylation, respectively. Transfection of Huh7 cells with the acetylation mimetic PCSK9–3KQ mimicked the effect of wild-type PCSK9 in reducing LDLR expression in Huh7 cells, while the deacetylation mimetic PCSK9–3KR failed to reduce LDLR protein expression (*Figure 4C*). Furthermore, transfection of the Huh7 cells with acetylation mimetic PCSK9–3KQ decreased the specific cellular binding and association ^125^I-LDL, whereas the transfection with deacetylation mimetic PCSK9–3KR did not affect the specific cellular binding and association ^125^I-LDL (*Figure 4D, 4E*). Importantly, deacetylation of rhPCSK9 by pre-incubation with rhSIRT1 in the presence of 5 mM NAD^+^ reduced the binding of rhPCSK9 to the EGF-A of LDLR (*Figure 4F*). These findings suggest that SIRT1-induced direct deacetylation reduces the activity of PCSK9 thereby allowing recycling of hepatic LDLR.

### 3.7 Human SIRT1 levels correlate inversely with PCSK9 and associate with reduced risk of MACE

To assess the translational value of our experimental findings, we assessed both human SIRT1 and PCSK9 plasma levels in patients presenting with ACS, an acute but common manifestation of ASCVD (see Supplementary material online, *Figure S9*). Interestingly, in line with our preclinical findings, an inverse correlation between baseline levels of SIRT1 and PCSK9 was observed (*R* = −0.1753, *P* = 0.0262) (see Supplementary material online, *Figure S10A*) which was particularly pronounced in patients with ST-elevation myocardial infarction (*r* = −0.2306, *P* = 0.0297). Among all patients, a total of 32 MACE (composite measure of non-fatal myocardial infarction, non-fatal stroke, ischaemia-driven revascularization, in-stent thrombosis, and death from cardiovascular causes) occurred during 1 year of follow-up, with Kaplan-Meier curves suggesting an inverse relationship between SIRT1 plasma levels and event-free survival (see Supplementary material online, *Figure S10B*). After adjustment for sex and age, patients in the highest SIRT1 tertile had a 64% reduced risk of MACE during 1 year after the index ACS [adjusted HR, 95% confidence interval (CI), 0.36, 0.15–0.88, *P* = 0.0242], which was similarly observed in multivariable analyses adjusting for smoking status, estimated glomerular filtration rate and presence of lipid-lowering therapies (adjusted HR, 95% CI, 0.26, 0.08–0.85, *P* = 0.0261) (see Supplementary material online, *Figure S10C*). Similar results were obtained in sensitivity analyses adjusting for sex, age, body mass index and a history of hypertension and/or diabetes (adjusted HR, 95% CI, 0.36, 0.14–0.90, *P* = 0.0288).

## 4. Discussion

Our data suggest that systemic SIRT1 replenishment by recombinant SIRT1 delivery decreases pro-atherosclerotic signatures, leading to diminished plaque burden in *ApoE^−/−^* mice. To the best of our knowledge, we show for the first time that PCSK9 acetylation is modified post-translationally, an essential step to maintain proper function i.e. directing LDLR towards lysosomal degradation. Further, we unveil SIRT1 treatment as a feasible and effective strategy to restore hepatic LDLR levels and thus increase LDL-C clearance by acting as a binding and consequently deacetylase partner of PCSK9. Finally, we show that in patients with ACS, SIRT1 expression levels correlate inversely with PCSK9 and associate with reduced risk of MACE. While the latter observation was independent from a variety of potential confounders, including but not limited to sex, age, kidney function and the use of lipid-lowering therapies, large-scale cohort studies are warranted to confirm these associations in independent settings and across patient subgroups.

Mounting evidence has demonstrated that intracellular activation of SIRT1 protects against atherosclerosis.^30–33^ Indeed, overexpression of *Sirt1* in endothelial cells of *ApoE^−/−^* mice fed on a high-fat diet promotes endothelium-dependent vasodilation and protects against atherosclerosis by increasing eNOS activity.^34^ Small molecule activators of intracellular SIRT1 reduced total cholesterol^35^ and especially plasma LDL-C levels.^17^ However, these studies provided limited mechanistic insight into the effects of SIRT1 on cholesterol metabolism and atherosclerosis. Recently, SIRT1 was measured in the circulation^19^; however, its function remains largely unknown. We here characterized the role of circulating SIRT1 using exogenous restoration of the reduced plasma protein levels in atherosclerotic mice. At the end of treatment, plasma levels of SIRT1 in *ApoE^−/−^* mice fed a high-cholesterol diet were similar to age-matched C57BL/6J wild-type controls. Recent studies showed an inverse correlation between the levels of circulating SIRT1 with plasma LDL-C and inflammatory cytokines.^19^ Indeed, we found that restoration of the reduced plasma SIRT1 levels in *ApoE^−/−^* atherosclerotic mice decreases plasma LDL-C levels, pro-inflammatory cytokines, and hence plaque burden.

The current monoclonal antibodies that target secreted PCSK9 do not alter the levels of atherogenic very low-density lipoprotein cholesterol (VLDL-C) in plasma.^36,37^ Elevated levels of LDL-C and VLDL-C are indeed associated with an increased risk of ASCVD. Recent studies show that PCSK9 plays an important role in the atherosclerosis progression beyond regulating the plasma LDL-C levels. PCSK9 modulates plaque formation and thrombosis by enhancing the production of pro-inflammatory cytokines including IL-6, IL-1β, TNF-α and IFN-γ.^38,39^ Thus, a combination of plasma lipid-lowering and anti-inflammatory treatment targeting PCSK9 reduction may protect against plaque inflammation.^40^ Although the current PCSK9 inhibitors lower lipid burden, their role in exerting consistent anti-inflammatory effects is inconclusive. Indeed, treatment with SIRT1 effectively reduced the levels of both atherogenic lipoproteins (LDL-C and VLDL-C) and pro-inflammatory cytokines, a crucial step in the prevention of ASCVD progression.

PCSK9 is synthesized as a 74 kDa proprotein and is subjected to autocatalytic cleavage in endoplasmic reticulum to form an ∼62 kDa mature protein. During its secretion from hepatocytes, PCSK9 undergoes several post-translational modifications including *N*-glycosylation, Tyr-sulfation, and phosphorylation.^16,41^ These post-translational modifications on PCSK9 affect its half-life and ability to bind and/or direct LDLR to hepatic lysosomes;^41^ this highlights the critical role of PCSK9-post-translational modifications in cholesterol homeostasis. Here, we report for the first time that PCSK9 undergoes a post-translational modification by acetylation. In fact, elevated acetyl-PCSK9 in plasma of atherosclerotic mice decreases the clearance of LDL-C as evidenced by enhanced degradation of hepatic LDLR. On the other hand, deacetylation of PCSK9 by circulating SIRT1 reduces its ability to degrade LDLR and hence aids in efficient clearance of LDL-C from plasma.

PCSK9 consists of a pro-domain, catalytic and C-terminal domains and is secreted as a complex containing the cleaved pro-domain non-covalently bound to the mature form.^15^ PCSK9 binds through the catalytic domain to the epidermal growth factor-like repeat (EGF-A) of LDLR on the cell surface in hepatocytes.^14,15^ This in turn increases the affinity of PCSK9 towards LDLR and increases plasma LDL-C levels in humans. The current injectable monoclonal antibody therapies block the interaction between PCSK9 and EGF-A of the LDLR on the hepatocyte cell surface, decrease plasma LDL-C levels.^42^ Of note, the levels of acetyl-PCSK9 were not changed in the hepatic tissue lysates, indicating that circulating, rather than intracellular SIRT1 exerts lipid-lowering and atheroprotective effects by acting as a novel binding and deacetylase partner of PCSK9 in the circulation.

We characterized the SIRT1-mediated deacetylation sites of PCSK9 using mass spectrometry, two within the catalytic domain (Lys243, Lys421) and one in the C-terminal domain (Lys506). Previous studies have shown that truncated PCSK9 containing only the pro-domain and catalytic domain could still bind to LDLR. However, the truncated PCSK9 without the C-terminal domain failed to degrade LDLR, indicating that domains in PCSK9 that are not required for binding are essential for PCSK9-mediated degradation of the LDLR. Indeed, transfection of hepatocytes with deacetylation mimetic (3KR) containing lysine sites in both catalytic and C-terminal domains failed to degrade LDLR, confirming that SIRT1-mediated deacetylation of PCSK9 is essential for its function to direct LDLR to lysosomal degradation. Further studies are envisaged to understand the effect of deacetylation on the conformation and itinerary of PCSK9 that leads to its reduced ability to interact with LDLR.

Our data suggest that restoration of systemic SIRT1 exerts atheroprotective effects through its interaction with PCSK9. Indeed, we provide translational evidence that SIRT1 acts as a novel binding partner of hepatic PCSK9 that inactivates protein function by deacetylation, thereby enhancing LDL-C clearance by upregulating hepatic LDLR expression. Finally, we show that high circulating levels of SIRT1 are associated with improved outcomes in patients with established ASCVD. Boosting systemic SIRT1 levels may be beneficial in regulating lipid metabolism and inflammation during ASCVD progression.

## 5. Limitations

Male mice were used in the current study due to the lack of notable effect of sex on the levels of circulating SIRT1.^43^ However, further studies are warranted to test the effect of boosting systemic SIRT1 levels in atherosclerotic female mice. Second, the concentration of recombinant SIRT1 injected in atherosclerotic mice was based on the results of *in vitro* experiments. Interestingly, in our current study we achieved a significant reduction in the LDL-C levels by boosting the circulating SIRT1 levels, additionally we also reduced the systemic inflammation which is still an unmet medical need with the current lipid-lowering drugs. Indeed, our future studies are designed to deliver SIRT1 directly to the liver to gain a longer therapeutic window and achieve target-specificity. Third, although a variety of potential confounders were considered in the multivariable-adjusted analyses, there are many other conditions that may influence SIRT1 plasma levels and event-free survival alike. While the consistent results across different regression models argue for high externally validity, large-scale observational data are warranted to assess the external validity of the herein reported findings across different patient groups. Indeed, owing to the limited sample size and to avoid model overfit, subgroup analyses could not be performed as part of this translational study. Finally, the acetylation/deacetylation ratio of PCSK9 in human samples was not provided as we could not detect acetylated lysine PCSK9 peptides in test cohorts without any prior enrichment for acetylated lysines. Our pilot study showed that pulling down of PCSK9 from plasma was required to detect the two PCSK9 isoforms, however, given the prospective design of the SPUM-ACS study, plasma volumes are limited, and acetylated/deacetylated ratios could not be assessed in this current study.

Translational perspective Although the current PCSK9 inhibitors lower lipid burden, they fail to exert consistent anti-inflammatory effects. Here, we unveiled boosting circulating SIRT1 levels as a feasible and effective strategy to increase plasma LDL-C clearance and decrease inflammation during atherosclerosis. Mechanistically, we showed for the first time that SIRT1 directly binds to PCSK9 and decreases its activity by deacetylation modification. We showed that higher levels of circulating SIRT1 are associated with reduced risk of major adverse cardiovascular events, thus establishing SIRT1 as a prognostic marker in patients with ASCVD. Targeting PCSK9 deacetylation through strategies that increase systemic SIRT1 levels could open exciting therapeutic avenues for the regulation of lipid metabolism and inflammation to eventually reduce the risk of ASCVD progression.

## Supplementary Material

cvaf087_Supplementary_Data

## Data Availability

The data underlying this article will be shared on reasonable request to the corresponding author.
