# Supplementary material for: Sirtuin-1 directly binds and deacetylates hepatic PCSK9 thereby promoting the inhibition of LDL receptor degradation
Source: Cardiovasc Res. 2025 Jul 14;121(9):1373–84. doi: 10.1093/cvr/cvaf087 (PMC12352300; doi:10.1093/cvr/cvaf087)
Supplement: cvaf087_Supplementary_Data [file cvaf087_supplementary_data.docx]

**Supplemental Material**

**Sirtuin-1 directly binds and deacetylates hepatic PCSK9
thereby promoting the inhibition of LDL receptor degradation**

Srividya Velagapudi PhD^1^, Melroy X. Miranda PhD^1^, Priyanka Adla MSc^2^, Simon Kraler MD^1^, Shafeeq Mohammed PhD^3^, Shekhar Baki MSc^2^, Jerome Robert PhD^4^, Lucia Rohrer PhD^4^, Hwan Lee PhD^5^, Hyun-Duk Jang PhD^5^, Slayman Obeid MD^6^, Anne Tailleux PhD^7^, Bart Staels PhD^7^, Naresh Babu V. Sepuri PhD^2^, Francesco Paneni MD^3^, Ravi Kumar Gutti PhD^2,^ Arnold von Eckardstein MD^4^, Hyo-Soo Kim MD^5^, Alexander Akhmedov PhD^1^, Giovanni G. Camici PhD^1^, Thomas F. Lüscher MD^1, 8, 9*^

^1^ Center for Molecular Cardiology, University of Zürich, Schlieren, Switzerland

^2^ Department of Biochemistry, School of Life Sciences, University of Hyderabad, Hyderabad, India

^3^ Center for Translational and Experimental Cardiology (CTEC), Department of Cardiology, Zurich University Hospital and University of Zürich, Zürich, Switzerland

^4^ Institute of clinical chemistry, University Hospital Zürich, Zürich, Switzerland

^5^ Biomedical Research Institute, Seoul National University Hospital, Seoul, S.Korea

^6^ Department of Cardiology, University Hospital Zürich, Zürich, Switzerland

^7^ Université Lille, INSERM, Institut Pasteur de Lille, Lille, France

^8^ Heart Division, Royal Brompton and Harefield Hospitals GSTT, UK

^9^ Cardiovascular Academic Group, King’s College, London, UK

^*^**Corresponding Author:** Thomas F. Lüscher MD, Center for Molecular Cardiology, University of Zürich, Wagistrasse 12, CH-8952, Schlieren, Switzerland and Heart Division, Royal Brompton and Harefield Hospitals GSTT and Cardiovascular Academic Group, King’s College, London, UK

TEL. +41 (0)44 250 40 80 | FAX +41 (0)44 250 40 90 | email: [thomas.luescher@zhh.ch](mailto:thomas.luescher@zhh.ch)

**Short Title:** Sirtuin-1 reduces PCSK9 activity by deacetylation

**Figure S1 – S10**

**Major Resources Table**

**Supplementary Figure 1**

**
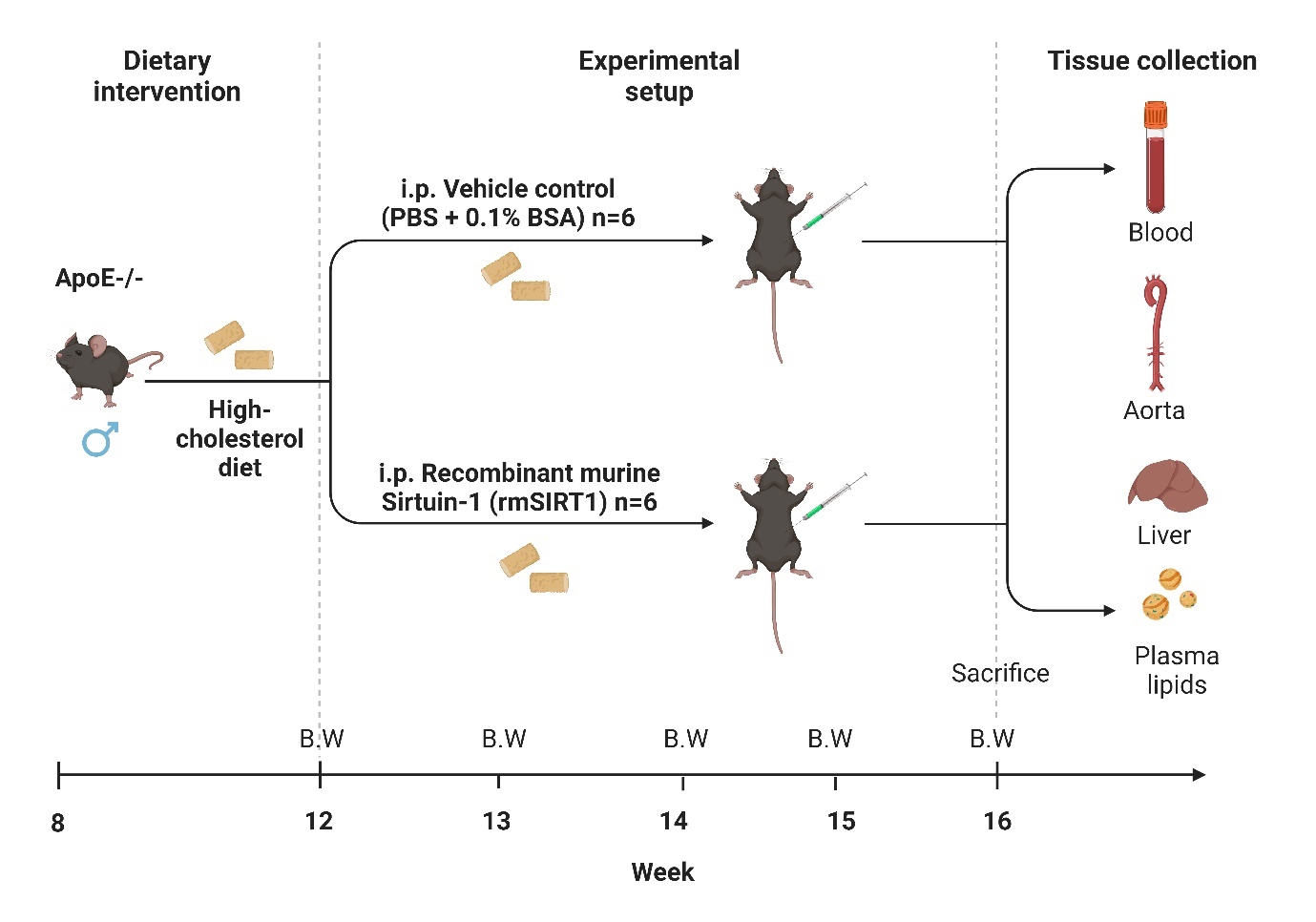
**

**Supplementary Figure 1. Experimental design of rmSIRT1 treatment in atherosclerotic *ApoE^-/-^* mice.** Eight-week-old *ApoE^-/-^* mice were fed on a high cholesterol diet (1.25% w/w) for 4 weeks and randomized to be treated with rmSIRT1 (n=6) or vehicle (Phosphate buffer saline containing 0.1% BSA) (n=6) for another 4 weeks. Created with BioRender.com

**Supplementary Figure 2**


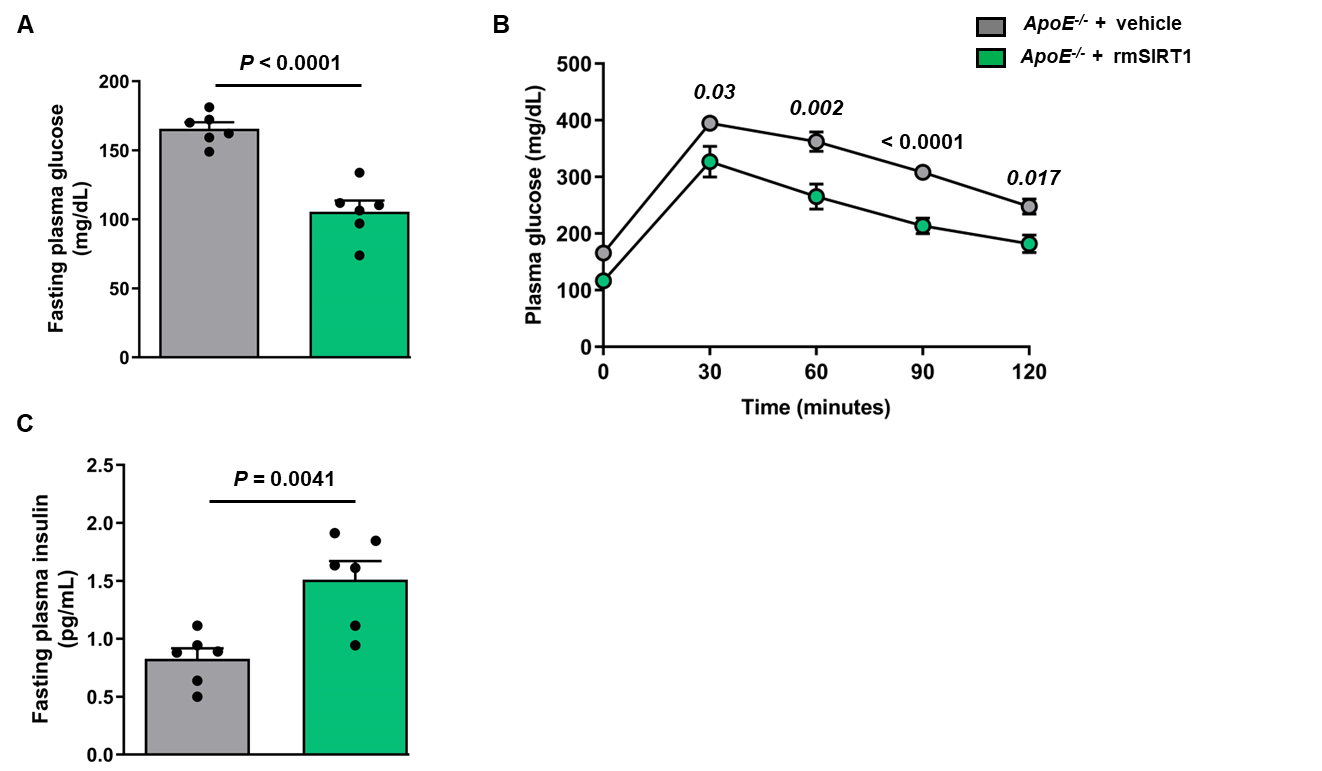


**Supplementary Figure 2. rmSIRT1 treatment improves glucose tolerance and insulin levels.** Eight-week-old *ApoE^-/-^* mice were fed on a high cholesterol diet (1.25% w/w) for 4 weeks and randomized to be treated with rmSIRT1 (n=6) or vehicle (PBS containing 0.1% BSA) (n=6) for another 4 weeks. **(A)** Plasma glucose levels measured in fasted (12 hours) mice. **(B)** Glucose tolerance test (GTT) was performed in mice fasted for 12 hours, and blood glucose was measured following 1 g/kg of body weight intraperitoneal glucose injection. **(C)** Serum samples collected during time point 0' was used to measure basal plasma insulin concentrations by mouse insulin ELISA. Values are represented as means ± SEM. Statistical significance was performed using Student’s unpaired t-test (A, C) or Two-way ANOVA with Holm-Sidak post-hoc test (B)

**Supplementary Figure 3**

**
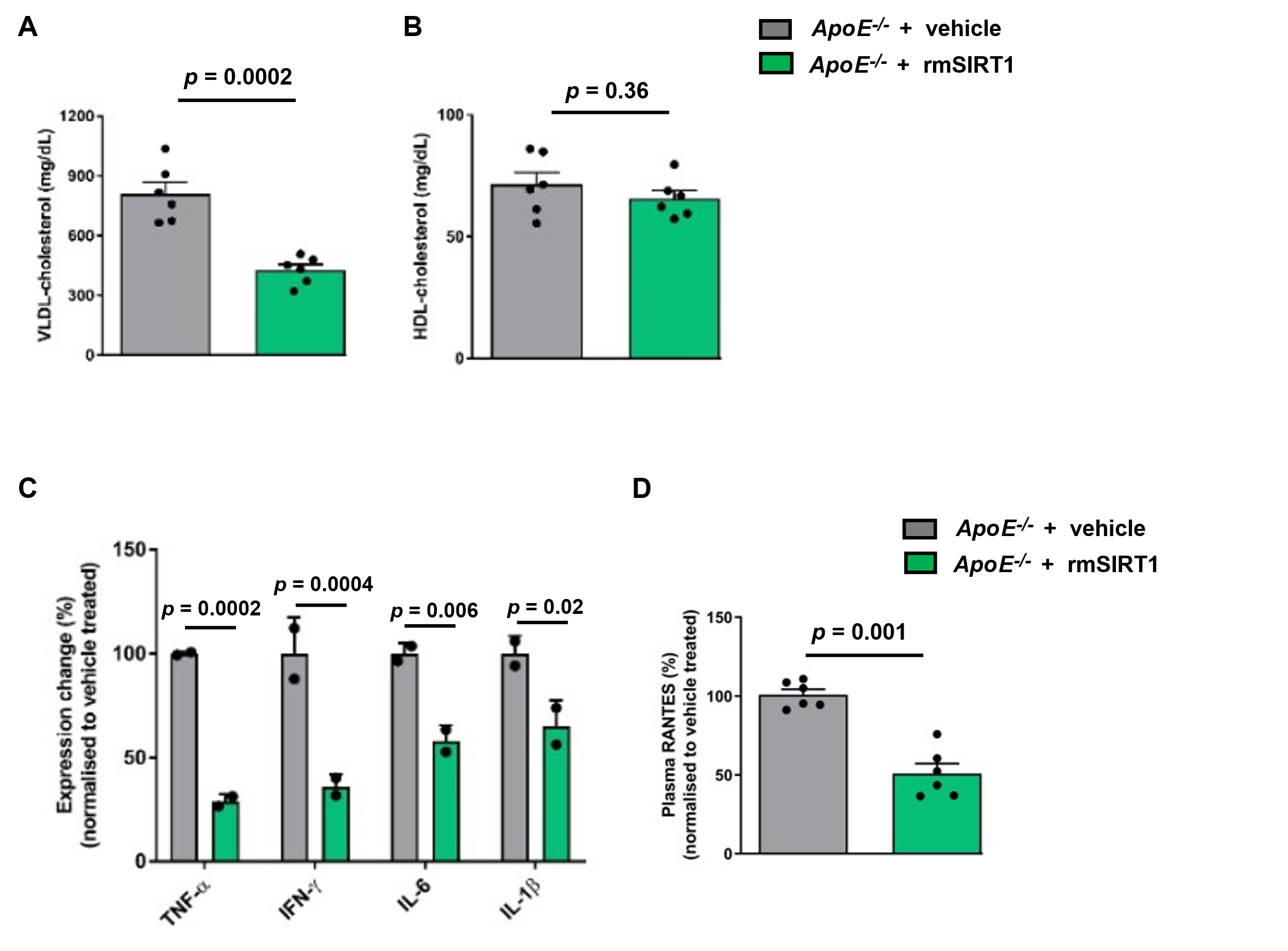
**

**Supplementary Figure 3. Circulating SIRT1 treatment reduces plasma VLDL-cholesterol and protects against atherosclerosis in *ApoE^-/-^* mice**. Eight-week-old *ApoE^-/-^* mice were fed on a high cholesterol diet (1.25% w/w) for 4-weeks and were randomized to be treated with rmSIRT1 (n=6) or with vehicle (PBS containing 0.1% BSA) (n=6) for another 4-weeks. **(A)** Bar graph of plasma VLDL-cholesterol and **(B)** HDL-cholesterol concentrations. VLDL – very low-density lipoprotein; HDL – high density lipoprotein. (**C**) Cytokine levels were measured using mouse cytokine array kit. Each run was performed by pooling 3 replicates per each group. The data represents average of two independent runs, (in total n=6). Data is normalised to the ApoE-/- mice treated with vehicle. **(D)** Bar graph of plasma RANTES/CCL5 measured using ELISA. Grey bars represent vehicle treatment and green bars represent rmSIRT1 treatment. *p-*values were calculated by Student’s unpaired two-sample t-test.

**Supplementary Figure 4**

**
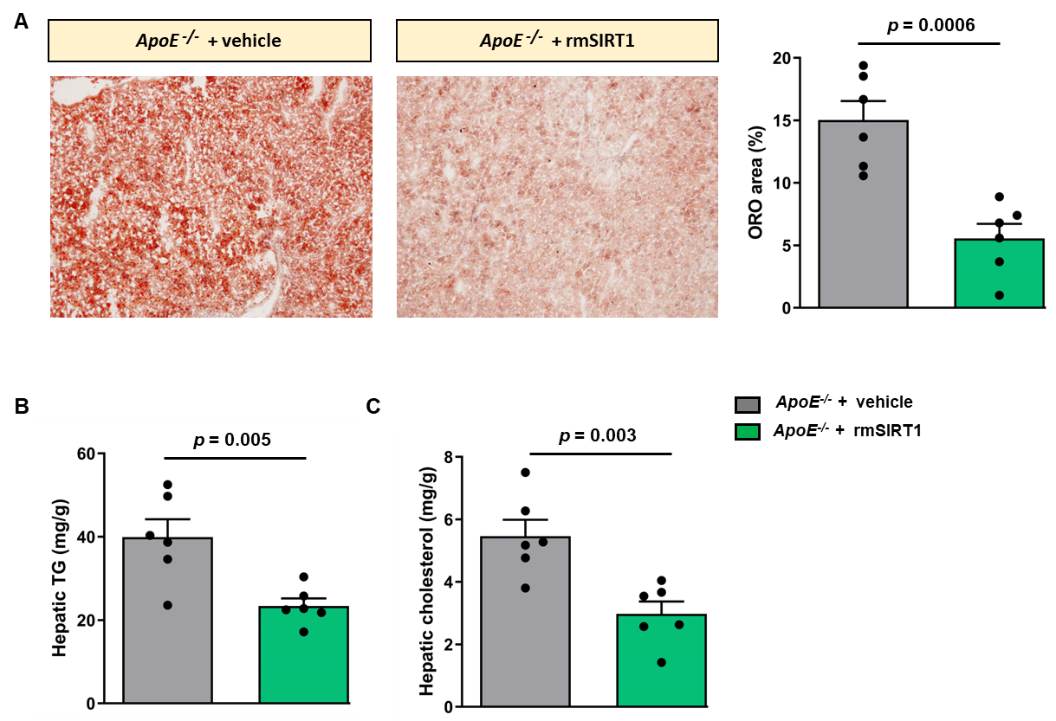
**

**Supplementary Figure 4. rmSIRT1 treatment decreases the hepatic steatosis phenotype**. Eight-week-old *ApoE^-/-^* mice were fed on a high cholesterol diet (1.25% w/w) for 4 weeks and randomized to be treated with rmSIRT1 (n=6) or vehicle (PBS containing 0.1% BSA) (n=6) for another 4 weeks. **(A)** Representative images of Oil Red-O staining and quantification, **(B)** hepatic triglycerides (TG) and **(C)** hepatic cholesterol. Grey bars represent vehicle treatment and green bars represent rmSIRT1 treatment. ORO, Oil-Red O. Values are represented as means ± SEM. Statistical significance was performed using Student’s unpaired t-test.

**Supplementary Figure 5**

**
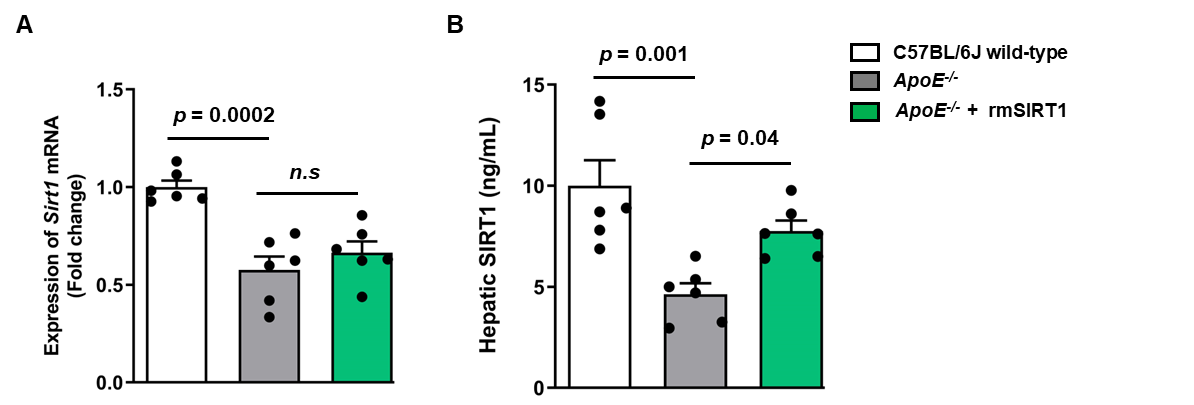
**

**Supplementary Figure 5. Hepatic expression of PCSK9 and SIRT1 in *ApoE^-/-^* mice**. **(A)** mRNA expression of *Sirt1* measured by qRT-PCR and **(B)** Protein level measured using SIRT1 ELISA in the hepatic tissue lysates of C57BL/6J mice (n=6) and ApoE^-/-^ mice on a high cholesterol diet (1.25% w/w) treated with vehicle (n=6) or with rmSIRT1 (n=6). Values are represented as means ± SEM. Statistical analysis was performed using one-way ANOVA test and significance determined by Tukey’s multiple comparison test.

**Supplementary Figure 6**

**
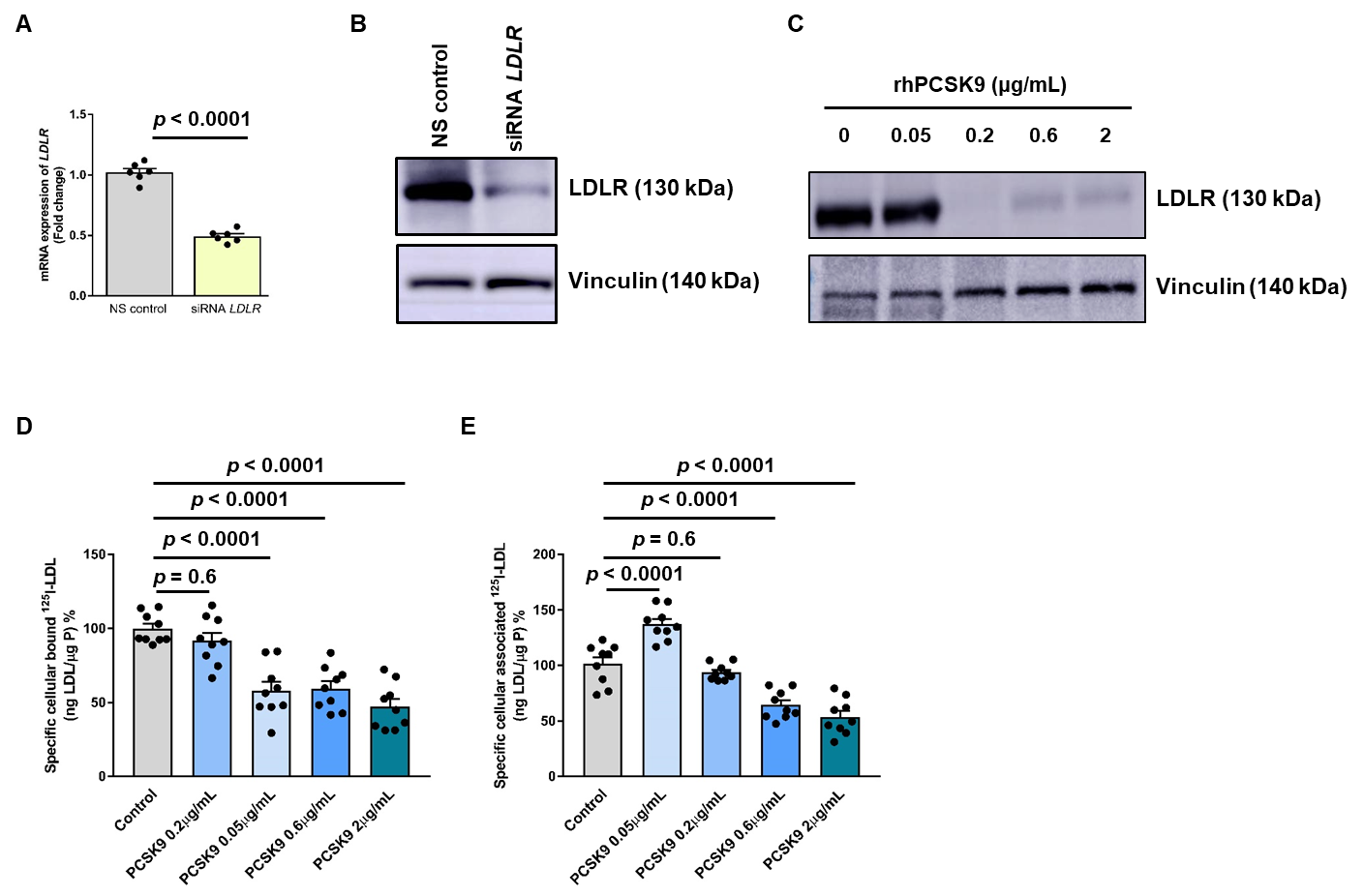
**

**Supplementary Figure 6. Transfection efficiency of LDLR and effect of exogenous PCSK9 treatment on LDLR levels in hepatocytes.** HuH-7 (hereafter Huh7) cells were transfected with a specific siRNA against LDLR or with non-silencing control siRNA (NS control). Assays were performed 72 hours post-transfection. **(A)** RT-PCR to assess knock-down efficiency of LDLR gene. *GAPDH* was used as housekeeping gene for normalization. **(B)** Representative LDLR (130 kDa) Western blot showing the efficacy of the silencing relative to the non-silencing siRNA (NS control) and Vinculin (140 kDa) used as the loading control. Huh7 cells were treated with rhPCSK9 for 2 hours at 37 ºC. **(C)** Western blot analysis of LDLR (130 kDa) degradation Huh7 cells upon exogenous rhPCSK9 treatment. Vinculin (140 kDa) used as the loading control. **(D)** Specific cellular binding of ^125^I-LDL was measured at 4ºC **(E)** Specific cellular association of ^125^I-LDL was measured at 37ºC**.** The results are represented as means ± SEM of three independent triplicate or more experiments (n=3). Statistical significance was performed using one-way ANOVA followed by Tukey’s multiple comparison test.

**Supplementary Figure 7**

**
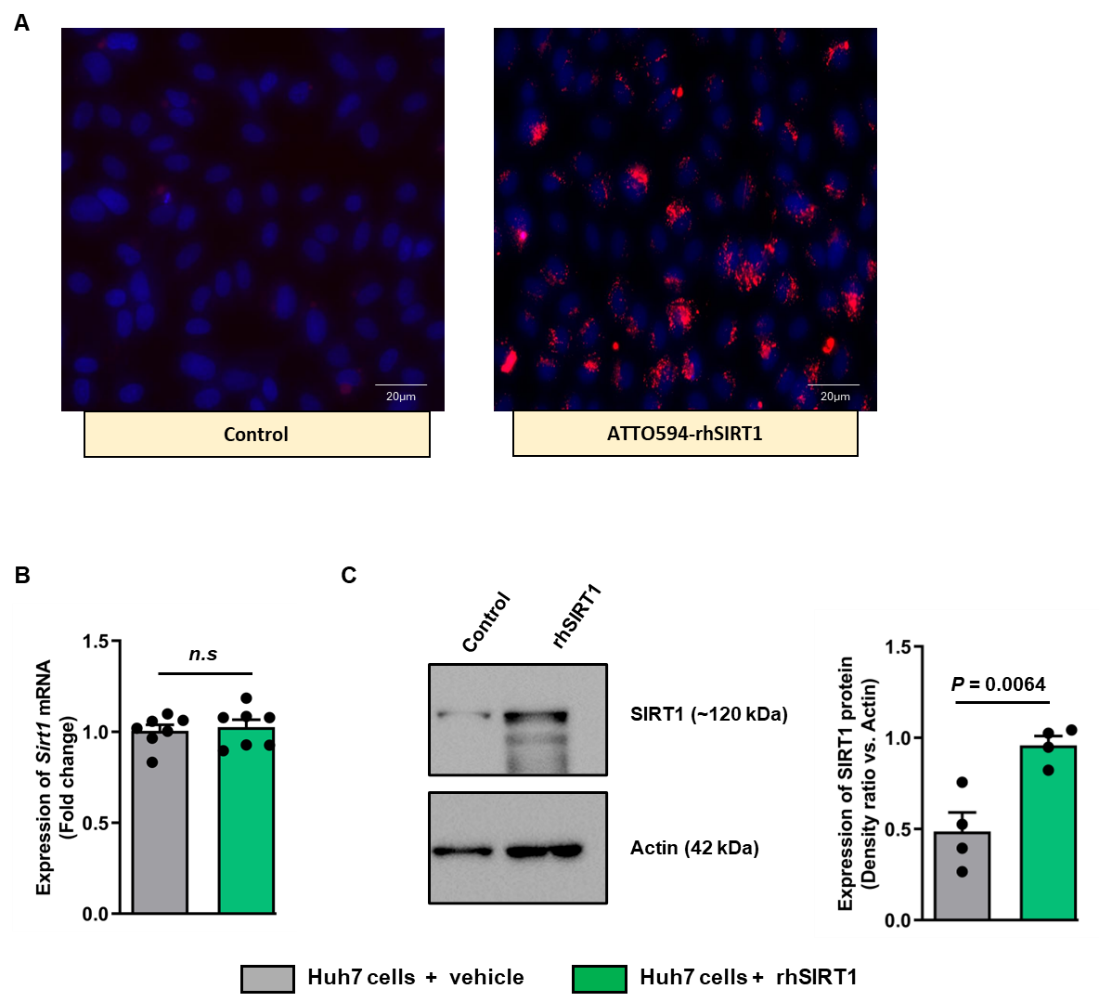
**

**
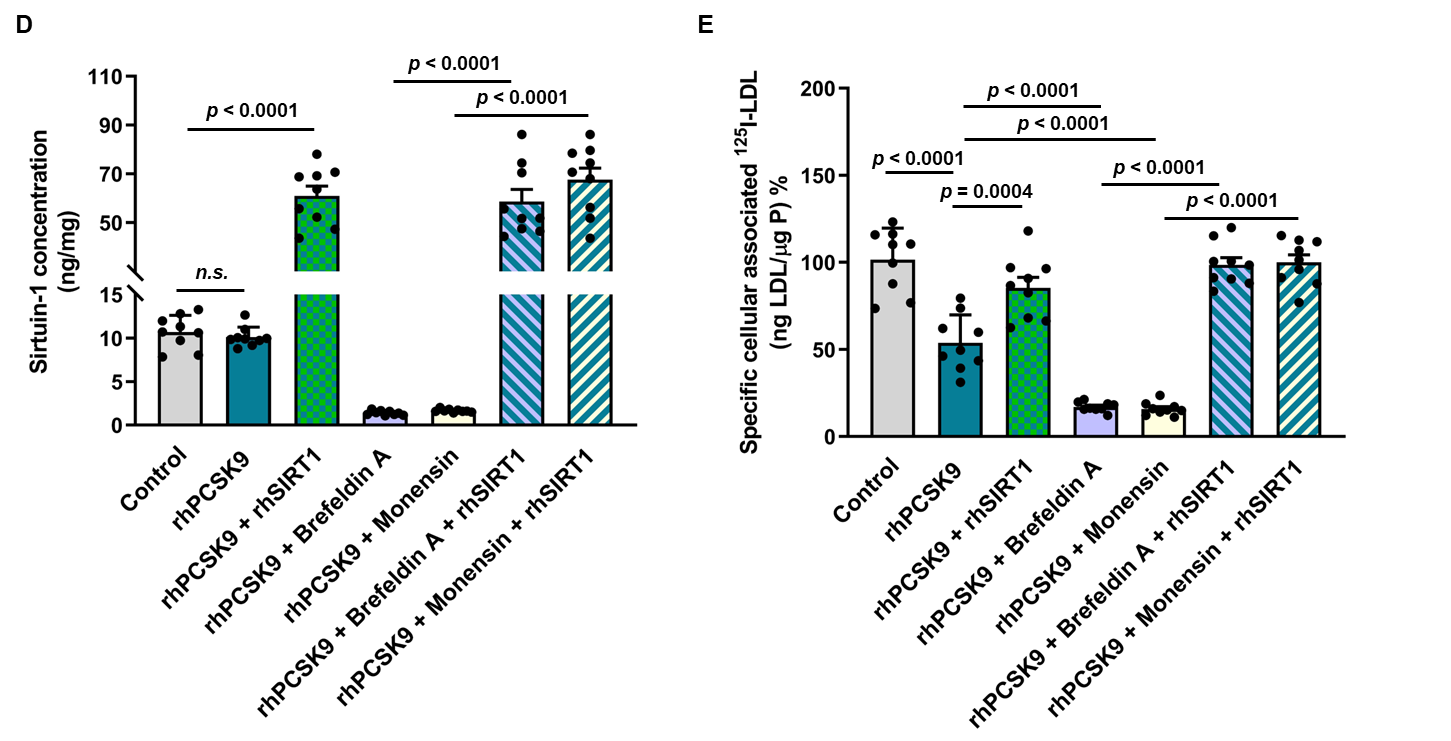
**

**Supplementary Figure 7. Secretion of SIRT1 is important for the cellular uptake of ^125^I-LDL.** **(A)** To study uptake of fluorescent labelled rhSIRT1, Huh7 cells were incubated with 1μmol/L of ATTO 594-rhSIRT1 for 2 hours at 37 ºC. Cells were fixed with 4%PFA (methods section), and images were obtained using widefield microscope. Nucleus stained with DAPI (in blue). Scale = 20μm. **(B-C)** Huh7 cells were incubated with rhSIRT1 overnight (~16 hours) and assayed by **(B)** gene expression analysed by qRT-PCR and **(C)** protein levels analysed by Western blotting analysis. Values are represented as means ± SEM of three independent experiments. Statistical significance was performed using Student’s unpaired t-test. **(D-E)** Where indicated Huh7 cells were treated with rhPCSK9 (2 μg/mL) or co-incubated with both rhPCSK9 (2 μg/mL) and rhSIRT1 (1 μmol/L) for 2 hours at 37ºC. Further the cells were treated with Brefeldin A (5 μg/mL) or Monensin (6 ng/mL). **(D)** Protein level measured using SIRT1 ELISA in the Huh7 cell lysates. **(E)** Specific cellular association of 125I-LDL was measured at 37ºC. Values are represented as means ± SEM of three independent triplicate or more experiments (n=3). Statistical significance was performed using one-way ANOVA followed by Tukey’s multiple comparison test.

**Supplementary Figure 8**

**
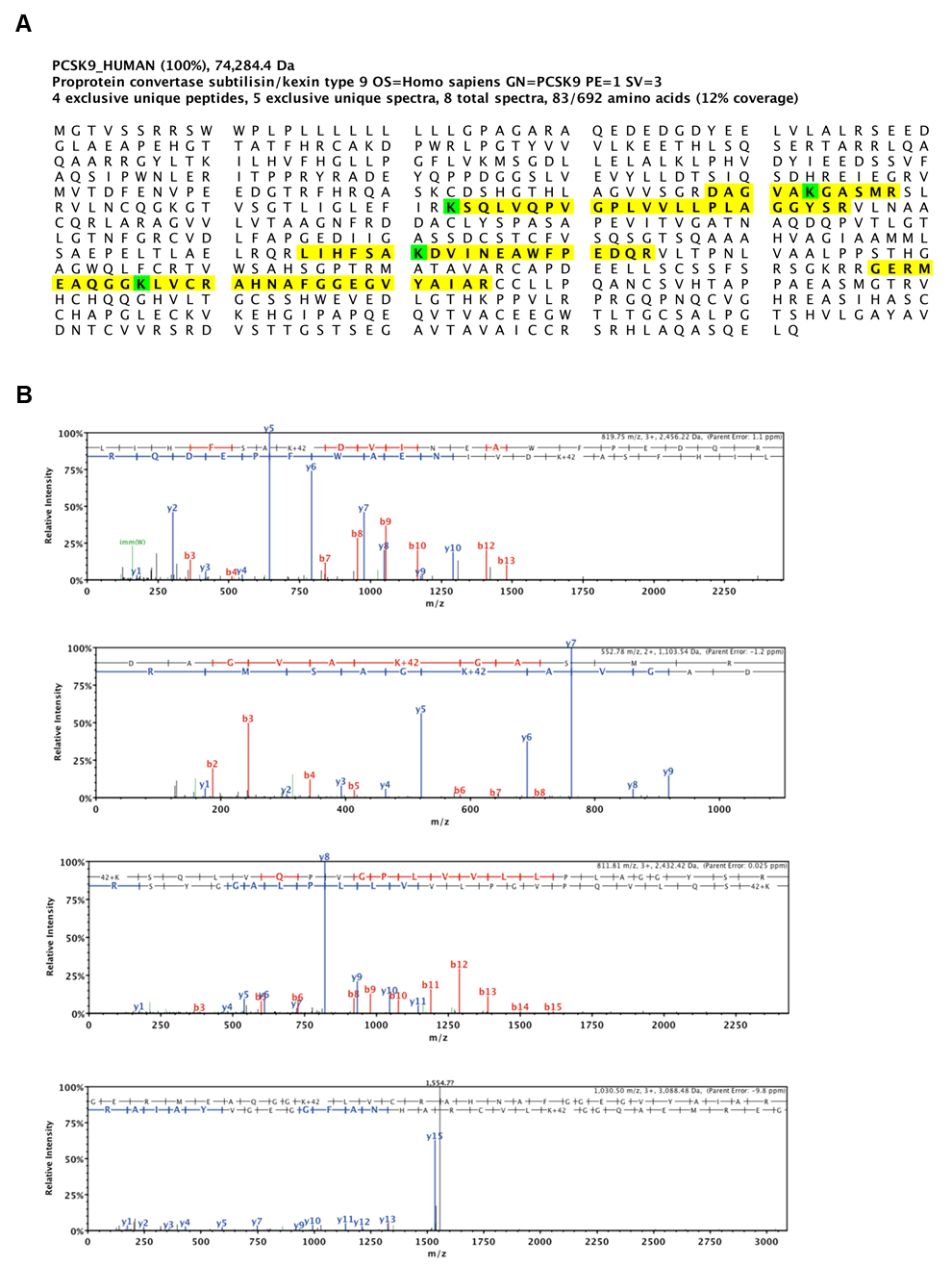
**

**Supplementary Figure 8. Identification and mapping of PCSK9 acetylation and deacetylation by SIRT1.** Huh7 cells were incubated with rhSIRT1 1μmol/L for 2 hours at 37ºC. The cell lysates were immunoprecipitated with anti-PCSK9 antibody and were further processed to perform Mass spectrometry analysis. The protein from Colloidal Coomassie stained band was digested with trypsin and the resulting peptides were analysed by LC-MS/MS and MALDI MS/MS. MS/MS data were analysed using the sequences of PCSK9 with the Mascot algorithm, allowing the detection of acetylated lysine residues. **(A)** MS/MS spectrum of all peptides recovered in the LC-MS/MS experiment for PCSK9, highlighted in yellow and lysine modification highlighted by green **(B)** Mass spectra obtained from the immunoprecipitated protein lysate digested with trypsin and subjected to LC-MS/MS analysis. MS/MS data were analyzed using the sequences of PCSK9 with the Mascot algorithm, allowing the detection of acetylated lysine residues (red). MS/MS spectrum of all peptides recovered in the LC-MS/MS experiment for PCSK9, highlighted in blue. Acetylation was detected on lysine residues 243, 273, 421 and 506 in the fragmentation spectrums of peptides from trypsin digested PCSK9. In the presence of rhSIRT1 treatment only lysine residue 273 was detected, but not of lysine residues 243, 421 and 506 in the fragmentation spectrums of peptides from trypsin-digested PCSK9.

**Supplementary Figure 9**

**
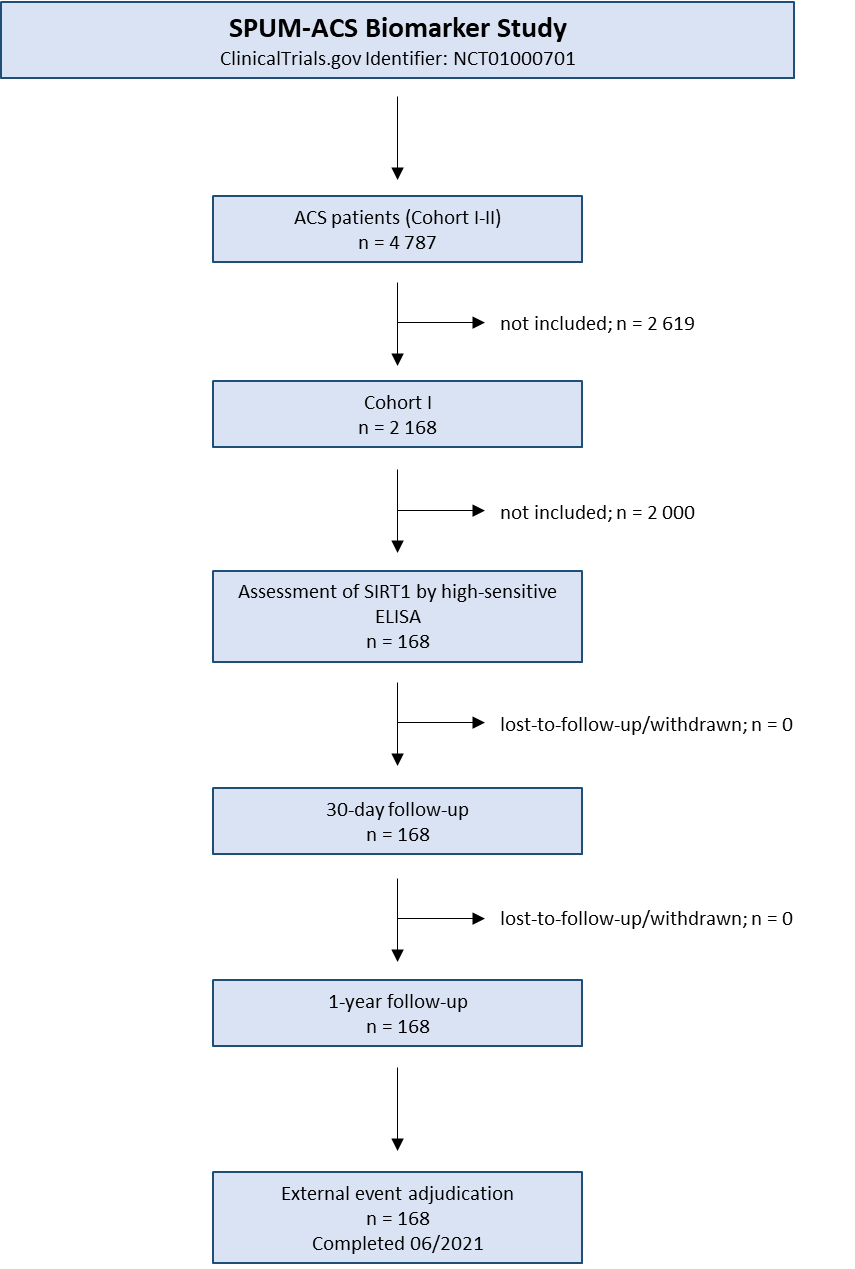
**

**Supplementary Figure 9.** **Flow chart of the study involving human ACS patients.**

**Supplementary Figure 10**

**
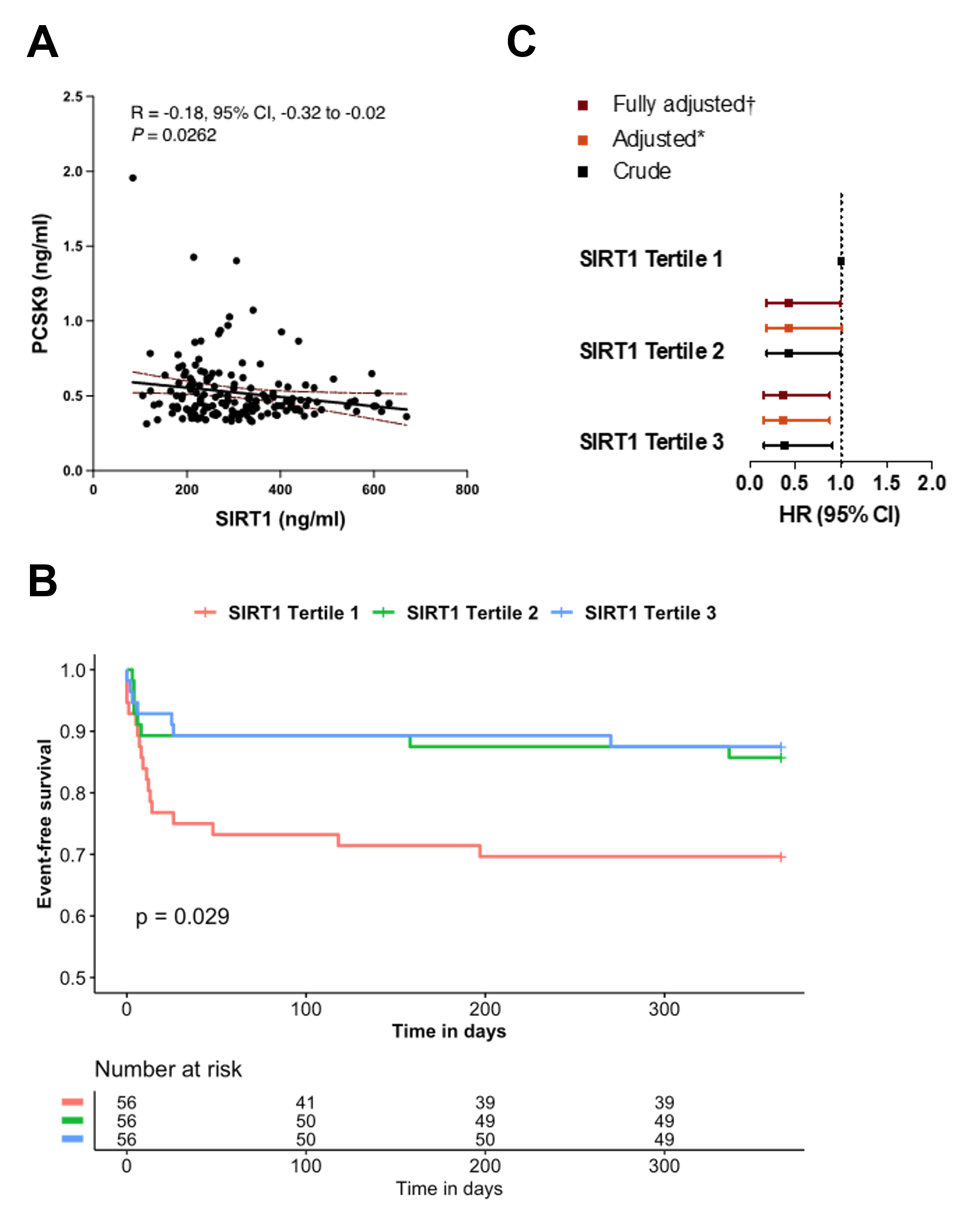
**

**Supplementary Figure 10. SIRT1 plasma levels in human patients with acute coronary syndromes.** (A) Correlation of plasma SIRT1 and PCSK9. (B) Kaplan-Meier curve for MACE-free survival per SIRT1 tertile along with log-rank-derived p value is shown. (C) Cox proportional hazard regression model derived risk estimates for major adverse cardiovascular events per SIRT1 tertile, with tertile 1 serving as the reference. **Adjusted for sex and age. †Additionally adjusted for estimated glomerular filtration rate, smoking status and presence of lipid-lowering therapies.*

**Major Resources Table**

**Animals (in vivo studies)**

| **Species** | **Vendor or Source** | **Background Strain** | **Sex** | **Persistent ID / URL** |
| --- | --- | --- | --- | --- |
| *ApoE^-/-^* | The Jackson Laboratories | C57BL/6 | M | https://www.jax.org/ |

**Antibodies**

| **Target antigen** | **Vendor or Source** | **Catalog #** | **Working concentration** | **Persistent ID / URL** |
| --- | --- | --- | --- | --- |
| LDLR | Abcam | ab52818 | 1:1000 | https://www.abcam.com/ldl-receptor-antibody-ep1553y-ab52818.html |
| hPCSK9 | Abcam | ab181142 | 1:1000 | https://www.abcam.com/pcsk9-antibody-epr76272-ab181142.html |
| mPCSK9 | R&D systems | AF3985 | 1:1000 | https://www.rndsystems.com/products/mouse-rat-proprotein-convertase-9-pcsk9-antibody_af3985 |
| Vinculin | Merck Millipore, Sigma Aldrich, Switzerland | V4505 | 1: 10,000 | https://www.sigmaaldrich.com/CH/de/product/sigma/v4505 |
| β-actin | Abcam | ab8226 | 1:2500 | https://www.abcam.com/beta-actin-antibody-mabcam-8226-loading-control-ab8226.html |
| acetyl-lysine | Cell signalling technology | 9441 | 1:1000 | https://www.cellsignal.com/products/primary-antibodies/acetylated-lysine-antibody/9441 |
| Goat Anti-Rabbit IgG(H+L)-HRP antibody | Southern Biotechnology, Birmingham, AL | 4050-05 | 1:10,000 | https://www.southernbiotech.com/goat-anti-rabbit-igg-h-l-mouse-human-ads-hrp-4050-05 |
| Goat Anti-Mouse IgG(H+L) | Southern Biotechnology, Birmingham, AL | 1031-05 | 1:10,000 | https://www.southernbiotech.com/goat-anti-mouse-igg-h-l-human-ads-hrp-1031-05 |
| IgG isotype control | Thermofisher Scientific | 02-6102 | the same  concentration  (20μg) of  the primary  antibody to be  used | https://www.thermofisher.com/antibody/product/Rabbit-IgG-Isotype-Control/02-6102 |
| CD68 | BioRad | MCA1957GA | 1:500 | https://www.bio-rad-antibodies.com/monoclonal/mouse-cd68-antibody-fa-11-mca1957.html?f=purified&JSESSIONID_STERLING=C031878C85A089A41519985D554A7545.ecommerce1&evCntryLang=CH-en&cntry=CH&thirdPartyCookieEnabled=true |

**Cultured Cells**

| **Name** | **Vendor or Source** | **Sex (F, M, or unknown)** | **Persistent ID / URL** |
| --- | --- | --- | --- |
| HuH7 cells | Japanese Collection or Research Bioresources  Cell Bank, JCRB Cell Bank, Osaka, Japan | M | CVCL_0336 |

| **Gene** | **Vendor or Source** | **Catalogue #** | **Final concentration** | **Vendor or Source** |
| --- | --- | --- | --- | --- |
| *LDLR* | Silencer Select, Thermo Fisher Scientific | s224006, s224007, s4 | 5nmol/L | https://www.thermofisher.com/ch/en/home/life-science/rnai/synthetic-rnai-analysis/ambion-silencer-select-sirnas.html |
| Non-silencing control siRNA | Silencer Select, Thermo Fisher Scientific | 4390843 | 5nmol/L | https://www.thermofisher.com/ch/en/home/life-science/rnai/synthetic-rnai-analysis/ambion-silencer-select-sirnas.html |
| Lipofectamine™  RNAiMAX  Transfection Reagent | Thermo Fisher  Scientific | 13778150 |  | https://www.thermofisher.com/order/catalog/product/13778150#/13778150 |
| Lipofectamine™ 3000  Transfection Reagent | Thermo Fisher  Scientific | L3000015 |  | https://www.thermofisher.com/order/catalog/product/L3000015#/L3000015 |

**Transfection Reagents and siRNAs**

**Primer Sequences**

| **Primer name** | **Sequence** | **Vendor or Source** |
| --- | --- | --- |
| h*LDLR* - forward | AAG GAC ACA GCA CAC AAC CA | Microsynth |
| h*LDLR* – reverse | CAT TTC CTC TGC CAG CAA CG | Microsynth |
| h*GAPDH* – forward | CCC ATG TTC GTC ATG GGT GT | Microsynth |
| h*GAPDH* - reverse | TGG TCA TGA GTC CTT CCA CGA TA | Microsynth |

**Other**

| **Description** | **Source / Repository** | **Persistent ID / URL** |
| --- | --- | --- |
| Animal diet (1.25% cholesterol) | D12108; Research Diets, USA | https://researchdiets.com/formulas/d12108c |
| Recombinant human PCSK9 | ab198471, Abcam | https://www.abcam.com/recombinant-human-pcsk9-protein-ab198471.html |
| Recombinant human Sirtuin-1 | CSB-EP822202HU, Cusabio Biotech, LubioScience, Switzerland | https://www.cusabio.com/Recombinant-Protein/Recombinant-Human-NAD-dependent-protein-deacetylase-sirtuin-1SIRT1-871066.html |
| Recombinant mouse Sirtuin-1 | CSB-EP846058MO, Cusabio Biotech, LubioScience, Switzerland | https://www.cusabio.com/Recombinant-Protein/Recombinant-Mouse-NAD-dependent-protein-deacetylase-sirtuin-1Sirt1-894910.html |
| PCSK9-LDLR in vitro binding assay kit | CY-8150, MBL International, Woburn, MA, USA | https://www.mblbio.com/bio/g/dtl/P/index.html?pcd=CY-8150 |
| Aminolink Plus immobilization kit | 44894, Pierce, Thermofisher Scientific | https://www.thermofisher.com/order/catalog/product/44894 |
| Protease and Phosphatase Inhibitor Mini Tablets,EDTA-free | A32961, Pierce, Thermofisher Scientific | https://www.thermofisher.com/order/catalog/product/A32961 |
| Pierce™ BCA Protein Assay Kit | 23225, Pierce, Thermofisher Scientific | https://www.thermofisher.com/order/catalog/product/23225 |
| Mouse SIRT1 ELISA | ab206983, Abcam | https://www.abcam.com/mouse-sirt1-elisa-kit-ab206983.html |
| Mouse PCSK9 ELISA | MPC900, R&D systems | https://www.rndsystems.com/products/mouse-proprotein-convertase-9-pcsk9-quantikine-elisa-kit_mpc900 |
| Human SIRT1 ELISA | LS-F6780, LS Bio | https://www.lsbio.com/elisakits/human-sirt1-sirtuin-1-sandwich-elisa-elisa-kit-ls-f6780/6780 |
| Human PCSK9 ELISA | DPC900, R&D systems | https://www.rndsystems.com/products/human-proprotein-convertase-9-pcsk9-quantikine-elisa-kit_dpc900 |

**ARRIVE GUIDELINES**

**Study Design**

| **Groups** | **Sex** | **Age** | **Number (prior to experiment)** | **Number (after termination)** | **Littermates**  **(Yes/No)** | **Other description** |
| --- | --- | --- | --- | --- | --- | --- |
| *ApoE^-/-^ +* vehicle control | M | 8-12 weeks (high fat diet); 12-16 week (vehicle control) | 6 | 6 | No |  |
| *ApoE^-/-^ +* rmSIRT1 | M | 8-12 weeks (high fat diet); 12-16 week (rmSIRT1) | 6 | 6 | No |  |

**Sample Size:** Sample size was estimated to achieve a power of 0.8 and α-level= 0.05 using a two-sided t test.

**Inclusion Criteria:** All the animals were included.

**Exclusion Criteria:** No animal was excluded.

**Randomization:** 12-week old *ApoE^-/-^* on a high-fat diet were randomly allocated to vehicle control and rmSIRT1 treatment groups.

**Blinding:** The treatment was blinded, i.e., person injecting the animals was unaware of the content in the injection.
